# Applications of Machine Learning in Human Microbiome Studies: A Review on Feature Selection, Biomarker Identification, Disease Prediction and Treatment

**DOI:** 10.3389/fmicb.2021.634511

**Published:** 2021-02-19

**Authors:** Laura Judith Marcos-Zambrano, Kanita Karaduzovic-Hadziabdic, Tatjana Loncar Turukalo, Piotr Przymus, Vladimir Trajkovik, Oliver Aasmets, Magali Berland, Aleksandra Gruca, Jasminka Hasic, Karel Hron, Thomas Klammsteiner, Mikhail Kolev, Leo Lahti, Marta B. Lopes, Victor Moreno, Irina Naskinova, Elin Org, Inês Paciência, Georgios Papoutsoglou, Rajesh Shigdel, Blaz Stres, Baiba Vilne, Malik Yousef, Eftim Zdravevski, Ioannis Tsamardinos, Enrique Carrillo de Santa Pau, Marcus J. Claesson, Isabel Moreno-Indias, Jaak Truu

**Affiliations:** ^1^Computational Biology Group, Precision Nutrition and Cancer Research Program, IMDEA Food Institute, Madrid, Spain; ^2^Faculty of Engineering and Natural Sciences, International University of Sarajevo, Sarajevo, Bosnia and Herzegovina; ^3^Faculty of Technical Sciences, University of Novi Sad, Novi Sad, Serbia; ^4^Faculty of Mathematics and Computer Science, Nicolaus Copernicus University, Toruń, Poland; ^5^Faculty of Computer Science and Engineering, Ss. Cyril and Methodius University, Skopje, North Macedonia; ^6^Institute of Genomics, Estonian Genome Centre, University of Tartu, Tartu, Estonia; ^7^Department of Biotechnology, Institute of Molecular and Cell Biology, University of Tartu, Tartu, Estonia; ^8^Université Paris-Saclay, INRAE, MGP, Jouy-en-Josas, France; ^9^Department of Computer Networks and Systems, Silesian University of Technology, Gliwice, Poland; ^10^University Sarajevo School of Science and Technology, Sarajevo, Bosnia and Herzegovina; ^11^Department of Mathematical Analysis and Applications of Mathematics, Palacký University, Olomouc, Czechia; ^12^Department of Microbiology, University of Innsbruck, Innsbruck, Austria; ^13^South West University “Neofit Rilski”, Blagoevgrad, Bulgaria; ^14^Department of Computing, University of Turku, Turku, Finland; ^15^NOVA Laboratory for Computer Science and Informatics (NOVA LINCS), FCT, UNL, Caparica, Portugal; ^16^Centro de Matemática e Aplicações (CMA), FCT, UNL, Caparica, Portugal; ^17^Oncology Data Analytics Program, Catalan Institute of Oncology (ICO) Barcelona, Spain; ^18^Colorectal Cancer Group, Institut de Recerca Biomedica de Bellvitge (IDIBELL), Barcelona, Spain; ^19^Consortium for Biomedical Research in Epidemiology and Public Health (CIBERESP), Barcelona, Spain; ^20^Department of Clinical Sciences, Faculty of Medicine, University of Barcelona, Barcelona, Spain; ^21^EPIUnit – Instituto de Saúde Pública da Universidade do Porto, Porto, Portugal; ^22^Department of Computer Science, University of Crete, Heraklion, Greece; ^23^Department of Clinical Science, University of Bergen, Bergen, Norway; ^24^Group for Microbiology and Microbial Biotechnology, Department of Animal Science, University of Ljubljana, Ljubljana, Slovenia; ^25^Bioinformatics Research Unit, Riga Stradins University, Riga, Latvia; ^26^Department of Information Systems, Zefat Academic College, Zefat, Israel; ^27^Galilee Digital Health Research Center (GDH), Zefat Academic College, Zefat, Israel; ^28^School of Microbiology & APC Microbiome Ireland, University College Cork, Cork, Ireland; ^29^Unidad de Gestión Clínica de Endocrinología y Nutrición, Instituto de Investigación Biomédica de Málaga (IBIMA), Hospital Clínico Universitario Virgen de la Victoria, Universidad de Málaga, Málaga, Spain; ^30^Centro de Investigación Biomédica en Red de Fisiopatología de la Obesidad y la Nutrición (CIBEROBN), Instituto de Salud Carlos III, Madrid, Spain; ^31^Institute of Molecular and Cell Biology, University of Tartu, Tartu, Estonia

**Keywords:** microbiome, machine learning, disease prediction, biomarker identification, feature selection

## Abstract

The number of microbiome-related studies has notably increased the availability of data on human microbiome composition and function. These studies provide the essential material to deeply explore host-microbiome associations and their relation to the development and progression of various complex diseases. Improved data-analytical tools are needed to exploit all information from these biological datasets, taking into account the peculiarities of microbiome data, i.e., compositional, heterogeneous and sparse nature of these datasets. The possibility of predicting host-phenotypes based on taxonomy-informed feature selection to establish an association between microbiome and predict disease states is beneficial for personalized medicine. In this regard, machine learning (ML) provides new insights into the development of models that can be used to predict outputs, such as classification and prediction in microbiology, infer host phenotypes to predict diseases and use microbial communities to stratify patients by their characterization of state-specific microbial signatures. Here we review the state-of-the-art ML methods and respective software applied in human microbiome studies, performed as part of the COST Action ML4Microbiome activities. This scoping review focuses on the application of ML in microbiome studies related to association and clinical use for diagnostics, prognostics, and therapeutics. Although the data presented here is more related to the bacterial community, many algorithms could be applied in general, regardless of the feature type. This literature and software review covering this broad topic is aligned with the scoping review methodology. The manual identification of data sources has been complemented with: (1) automated publication search through digital libraries of the three major publishers using natural language processing (NLP) Toolkit, and (2) an automated identification of relevant software repositories on GitHub and ranking of the related research papers relying on learning to rank approach.

## Introduction

The human microbiome represents a complex community of trillions of microorganisms (bacteria, archaea, viruses, as well as microbial eukaryotes such as fungi, protozoa and helminths), well-known to affect general health and homeostasis, e.g., by actively participating in human metabolism and regulating the immune system. Several disease-related states have been linked with a disruption of the steady relationship between the gut microbiota and gut epithelial cells (dysbiosis) (Petersen and Round, 2014). In the last decade, the number of microbiome-related studies has increased notably, and big populational studies such the Human Microbiome Project (Human Microbiome Project Consortium, 2012), the metagenomics of the Human Intestinal Tract (Qin et al., 2010), and the American Gut Project (McDonald et al., 2018), among others, have considerably increased the available data on human microbiome composition and function. These studies provide the essential material to deeply explore host-microbiome associations and their relation to the development and progression of various complex diseases.

Most of the above-mentioned data were generated by amplicon sequencing, primarily by profiling the V3-V4 region of the 16S rRNA marker gene, which allows taxonomic identification of bacteria and archaea. A smaller number of studies have also used 18S rRNA marker gene sequencing to study the microbial eukaryotes such as fungi and protozoa (Elekwachi et al., 2017; Yarza et al., 2017). In both cases, amplicon sequences exhibiting a predefined level of sequence similarity (usually 97%) are commonly clustered into Operational Taxonomic Units (OTUs) that represent the abundance of a particular bacterial taxon (Blaxter et al., 2005). However, due to recent advances in high-throughput sequencing technologies, OTUs are increasingly being replaced by amplicon sequence variants (ASVs), which are un-clustered error-corrected reads (Callahan et al., 2017). After clustering (in case of OTUs) or denoising (in case of ASVs) and feature classification and annotation, the OTU/ASV table with the correspondent abundances is generated. Despite the cost-effective nature of this methodology, 16S rRNA gene sequencing has some drawbacks, e.g., (i) reliable bacterial classification is mostly possible down to the genus level (Winand et al., 2020); and (ii) limited information of the bacterial genes and their functions is obtained.

Another approach that is increasingly being used is the shotgun sequencing of microbial DNA without selecting a particular gene. This approach allows for more accurate classification of the microbial communities (even down to the strain level), and also permits the study of genes and their functions, e.g., by the construction of Gene Ontology (GO) (Ashburner et al., 2000) tables and Kyoto Encyclopedia of Genes and Genomes (KEGG) (Kanehisa and Goto, 2000) pathways (Scholz et al., 2016).

Improved data-analytical tools are needed to exploit all the information from these biological datasets, considering the peculiarities of microbiome data, i.e., compositional data, heterogeneous and sparse nature of the datasets. The possibility of predicting host-phenotypes based on taxonomy-informed feature selection to establish an association between the microbiome, predict various disease states or improve human health is beneficial for personalized medicine. In fact, the gut microbiome has become an integral part of personalized medicine, as it not only significantly contributes to inter-individual variability in health and disease, but also represents a potentially modifiable factor that can be targeted by therapeutics in a personalized manner (Kashyap et al., 2017). In this regard, ML may provide new insights into biomedical analyses, by the development of models that can be used to predict outputs such as categorical labels, binary responses, or continuous values.

Recently, a number of studies have applied ML techniques to analyze human microbiome data, harvesting the hidden knowledge to uncover and understand diversity in taxonomy and function within microbial communities and their impacts on human health. Firstly, to support the taxonomic representation and differentiation in microbiology, models were developed to support the classification of microbial features (Cai and Sun, 2011; Bonder et al., 2012; Werner et al., 2012; Vervier et al., 2016). Secondly, ML was used for the inference of host phenotypes in disease prediction (Pasolli et al., 2016; Flemer et al., 2017; Asgari et al., 2019; LaPierre et al., 2019; Thomas et al., 2019), and finally, to support the use of microbial communities to stratify patients by the characterization of state-specific microbial signatures (Koohi-Moghadam et al., 2019; Wirbel et al., 2019; Yachida et al., 2019).

Here, we aim to review the application of the different ML techniques to human microbiome data analysis and the available ML-based software resources currently used in the analysis of human microbiome data. The review is mainly focused on the application of ML in microbiome studies related to causality and clinical use for diagnostics, prognostics, and therapeutics.

## Methods

### Scoping Review Methodology – Identification, Selection, and Organization of Relevant Publications

This study follows the scoping review methodology for searching and assessment of the relevant studies (Arksey and O’Malley, 2005). The breadth of the ML methodology and data types in ML-based microbiome analysis hinder the thorough qualitative analyses of the selected papers, thus giving a scoping nature to this review which aims to search, select and synthesize the findings related to the application of ML in microbiome analysis and identify the available research evidence. The scientific methodology of all emerging review types is common as they rely on a formal and explicit methods for search, selection and evaluation of published studies (Moher et al., 2015). An example of such thorough review guidelines is Preferred Reporting Items for Systematic Review and Meta-Analysis (PRISMA) for systematic reviews in healthcare (Moher et al., 2010). The methodological framework for scoping reviews is established following the exact way how systematic reviews are conducted, providing sufficient details to reproduce the results (Moher et al., 2015). The workflow for a scoping review and adopted in this study, includes 5 stages (Arksey and O’Malley, 2005): (1) identification of a research question; (2) identification of relevant studies; (3) study selection; (4) charting the data; (5) collating, summarizing, and reporting the results.

As the motivation and relevance of the research question has already been extensively elaborated, we focus here on the methodology used to identify and select relevant studies. We have used both manual and automated search of literature corpus in the identification step, performing three independent processes:

• Manual search – crowdsourcing of the studies relevant for the review topic by all members of the COST Action CA18131 “Statistical and machine learning techniques in human microbiome studies”. In this way, in total 54 papers were collected, and 35 papers are included in the final list.
• An automated search of digital libraries of three major publishers (PubMed, Springer and IEEE) using NLP Toolkit (Zdravevski et al., 2019) to automate the literature search, scanning, and eligibility assessment. This automated search was additionally constrained to the period from January 2008 to December 2019 (and including those). In total 5,935 papers were identified using this method, after removal of duplicates that appear as a result of multiple searches using the similar subsets of keywords. From that, 67 papers were selected for a manual check, and 37 papers are included in the final list.
• An automated search through the available GitHub resources using NLP algorithms to identify relevant software repositories and extract corresponding scientific papers. The papers were automatically ranked by relevance using the pointwise learning to rank approach (Fejzer et al. unpublished) trained using the manually collected and labeled papers. We found 357 repositories that matched human microbiome research (within 1339 matching microbiome research). In these locations, we found 410 papers, and based on model score, selected 29 papers. The final list includes 17 papers.

The study selection procedure comprised scanning and eligibility assessment steps. The scanning was used in NLP Toolkit thread and served to remove the duplicates and exclude the papers whose title and abstract could not be analyzed due to unavailability, parsing errors, or any other reason. The eligibility assessment step referred to all identified studies in order to select only those relevant for this review. For the studies identified by the NLP Toolkit, the relevance of the study was assessed based on the NLP augmented evaluation of title and abstract according to the prespecified criteria. The papers identified through an automated search of GitHub resources were scored for relevance using the trained model based on learning to rank approach. The detailed description of the methodology used in automated search and eligibility assessment for both NLP Toolkit and learning to rank approach are provided in Supplementary Material.

**FIGURE 1:**
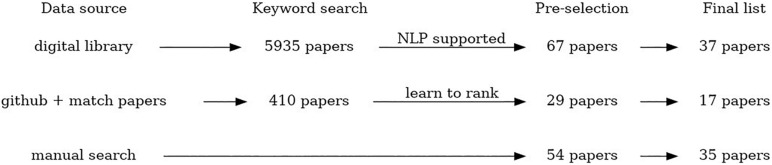
The scheme summarizing the process of paper selection for this review.

The scoping review workflow illustrating the number of identified, scanned, and articles included in this scoping review using all three data collection procedures is presented in Figure 1. The listing of all articles included in this study labeled with respect to different descriptors/keywords is available as Multimedia Appendix.

### Medical Subject Headings Annotations

Medical Subject Headings (MeSH) is the NLM controlled vocabulary thesaurus used for the indexing of articles in PubMed. We have used this resource to catalog the 89 papers included in this review from a biomedical perspective to explore the areas that are implementing ML techniques in human microbiome studies. The Wordclouds tool was used to summarize the information^1^.

### Data Acquisition From Different Resources

The human microbiome has been described as a fingerprint, unique and specific to each individual, set in early life and modeled by diet, lifestyle and environmental factors (Gilbert et al., 2018). Besides the high inter-variability of the microbiome, there are some shared functions between the different microbial strains, the so-called core human metagenome established by the analysis of large population studies. Moreover, the characterization of the microbial genes implied in human metabolic functions, the creation of a “gene catalog” of the human microbiome, and the description of differences between specific human conditions have been pointed out by assessing populational studies that have generated great amounts of metagenomics data. The list of main population studies, gene catalogs generated and database resources for analyzing microbiome data, respectively, are shown in Table 1.

**TABLE 1:** Different resources and databases for microbiome data acquisition.

Study name	Samples	Description	Data Availability/Ref.
Human Microbiome Project phase 1 and 2 (HMP1, iHMP or HMP2).	HMP1: Healthy adult population of 242 individuals. Samples from 35 body sites, retrieving 13,572 samples in total (i.e., feces = 2,151; buccal mucosa = 633; vagina = 551; other body sites = 10,237). HMP2: Data related to three main conditions: preterm birth, diabetes, and inflammatory bowel disease.	The project generated a huge number of nucleotide sequences of microorganisms, by simultaneously creating protocols to promote reproducible sampling and data generation in microbiome studies, essential for the establishment of computational methods for microbiome data analysis. The iHMP aimed to study host-microbiome interactions by joining the analysis of the immunity, metabolism, and molecular activity to untangle the complex interplay between the host and its microbiomes.	https://hmpdacc.org/.
Metagenomics of the Human Intestinal Tract (MetaHIT)	Overweight and obese adults, and patients with IBD from Spain and Denmark.	This project aimed to characterize the human gut metagenomes of healthy, overweight and obese adults, and patients with IBD from Spain and Denmark. The project has generated 576.7 Gb of sequence and predicted 3.3 million unique open reading frames (ORFs).	Li et al., 2014
American Gut (AGP).	“Wild-type” population. This project currently included microbial sequences from 15,096 samples of 11,336 human participants.	Initially, it was designed to study the North American population, but the initiative attracted also people from the United Kingdom, Australia and other countries. People volunteered to collect feces and fill a questionnaire of their general health status, disease history, and lifestyle to get their microbiome sequenced. The diversity of the data, and the high number of microbial sequences allowed to classify the microbiomes into four great categories, and differences according to country, sex, age, and race, were observed, moreover it adds up to ∼467 million of 16S rRNA V4 gene fragments.	http://americangut.org
The Integrated Gene Catalogs 1 and 2 (IGC, and IGC2).	Comprises more than nine million genes observed in gut microbes. Recently, an updated version of the catalog, denoted added 517,488 supplementary genes.	A catalog of microbial genes including important functions for host-bacterial interaction, and the determination of the so-called “minimal gut bacterial genome” that encompasses genes from bacteria found in most human guts. It has been applied successfully to study host-microbiome associations in the context of different diseases such as type 2 diabetes, obesity, and other pathologies. Genes with co-varying abundance levels can be clustered (Nielsen et al., 2014; Plaza Oñate et al., 2019) to allow taxonomic and functional profiling, and reveal potential disease markers in metagenome-wide association studies.	Qin et al., 2010; Wen et al., 2017
The Unified Human Gastrointestinal Genome (UHGG) and Protein (UHGP) catalogs.	286,997 microbial genomes from the available human gut microbiome datasets.	These catalogs were created by analyzing 286,997 microbial genomes and over 625 million protein sequences, including more than four thousand species. Up to 71% of the taxons analyzed are viable but non-culturable (VBNC), lacking viable culture indicating that most of the microbial diversity in the catalog remains to be characterized.	Almeida et al., 2021
MGnify (formerly EBI Metagenomics)	Diverse microbiome types, including ∼63.000 samples from human microbiome.	Free-access resource for browsing, analyzing, and archiving metagenomic and metatranscriptomic data. The platform contains an automated pipeline for the analysis of microbiome data to determine the taxonomic diversity along with functional and metabolic characteristics.	https://www.ebi.ac.uk/metagenomics/ Mitchell et al., 2018, 2020
CuratedMetagenomeData	Includes taxonomic and metabolic functional profiles and curated metadata for the publicly available human microbiome samples generated by shotgun metagenomic sequencing.	Bioconductor (Gentleman et al., 2004) package that provides uniformly processed and manually annotated human microbiome data. All data is processed by using the same pipeline, i.e., MetaPhlAn2 (Truong et al., 2015) for taxonomic abundance, gene marker presence and absence, and HUMAnN2 (Franzosa et al., 2018) for coverage and abundance of metabolic pathways and gene families abundance.	Pasolli et al., 2017
Qiita	Sequencing, proteomics, taxonomic, transcriptomics, and metabolomics data.	Open-source management platform for microbial studies. It integrates different omics data, providing a database and compute resources for the analyses of microbiome data.	https://qiita.ucsd.edu/ Gonzalez et al., 2018
ML Repo	15 published human microbiome datasets.	Public web-based repository of 33 curated classification and regression tasks from 15 published human microbiome datasets. Therefore, it is not only the data repository but it can also be used for benchmarking new machine learning approaches for microbiome data analyses.	https://knights-lab.github.io/MLRepo/ Vangay et al., 2019

### Data Selection and Pre-processing for ML-Based Applications

Proper normalization of microbiome data is essential for obtaining relevant outcomes from their further processing (Weiss et al., 2017) including ML techniques, with the primary aim to ensure comparability of data across samples. The issue is the large variability in library sizes, constrained additionally by the maximum number of sequence reads of the instrument. This total count constraint induces strong dependencies among the abundances of the different taxa; an increase in the abundance of one taxon requires the decrease of the observed number of counts for some of the other taxa so that the total number of counts does not exceed the specified sequencing depth (Rivera-Pinto et al., 2018). Moreover, observed raw abundances and the total number of reads per sample are non-informative since extracted DNA was normalized during library preparation and also, they represent only a fraction or random sample of the original DNA content in the environment. While Weiss et al. (2017) proposed normalization strategies like cumulative sum scaling, variance stabilization, and trimmed-mean by M-values, none of them really captures the above property of scale invariance, known from the concept of compositional data as observations carrying relative information (Aitchison, 1986; Pawlowsky-Glahn et al., 2015; Filzmoser et al., 2018). A very simple approach of normalization to the total amount of extractable microbial DNA or the total number of targeted cells counted by either flow cytometry or qPCR represented a step in the right direction.

The main idea is to represent the original microbiome (compositional) data in new variables, formed by interpretable log-ratios or their aggregates (log-contrasts), and then to continue in standard statistical or ML processing. There is an increasing number of publications motivating and using the log-ratio methodology of compositional data for statistical processing of microbiome (e.g., Gloor et al., 2017; Silverman et al., 2017; Quinn et al., 2018; Randolph et al., 2018; Rivera-Pinto et al., 2018; Jiang et al., 2019; Quinn and Erb, 2020). However, it still cannot be considered as a mainstream concept in microbiome analysis, mostly due to the high dimensionality of samples and the necessity of dealing with (count) zeros. From the perspective of ML techniques, the outcome is not necessarily a better classification, this depends, as usual, on the capability of a specific method to extract information from (transformed) data, but the compositional approach should reveal relevant sources of differences (microbiome markers) among microbiome samples or groups of samples (e.g., diseased vs healthy).

### Literature Review of ML Applications for Microbiome Studies

We finally selected 89 papers for review (35 manually selected, 37 using the automated NLP Toolkit search through PubMed, IEEE Xplore and Springer digital libraries, and 17 by searching in GitHub repositories). ML implies training and evaluation of models to identify, classify, and predict patterns from data. Unsupervised methods aim to identify plausible patterns in the data, without the use of ground truth/labels, while supervised approaches rely on the given labels to train the model and learn the mapping of input features to the labels at the output.

Here, we present the most frequently applied ML methods in microbiome studies, taking into account that ML applied on the large volumes of microbiome data can offer valuable insight into human-microbiome interactions We focused on those studies in which ML is used for: (i) the classification and prediction of microbial taxa, i.e., microbial classification and taxonomic assignment; (ii) the prediction of the host phenotype by linking microbial populations to phenotypes and ecological environments, i.e., disease prediction, and (iii) the usage of microbial communities for understanding disease mechanisms, and the further application in personalized medicine (companion test), i.e., biomarker-finding.

Finally, many of the reviewed ML methods have implemented within the Bioconductor packages, initially developed for the microchip/microarray-based data analyses (Gentleman et al., 2004). Consequently, the lessons learned enabled their integration into web portals, such as Microbiome Analyst^2^ (Chong et al., 2020) for a comprehensive statistical, visual and meta-analysis of microbiome data.

#### Supervised Learning Methods

Supervised learning trains and evaluates the model based on the input data complemented with ground truth/labels indicating the outcomes for the given input samples. Common supervised learning approaches include regression analysis and statistical classification.

##### Logistic regression

Logistic regression (LR) is a statistical method that learns a model that predicts an outcome for a binary variable, Y, from one or more response variables categorical or continuous, X. (Hoffman, 2019).

Logistic regression has been used for establishing microbial signatures in bacterial vaginosis (Beck and Foster, 2015), a disease associated with the vagina microbiome, however, no single microbe has been found to cause it. The authors found that both classifiers identify largely similar microbial community features and that only a few features were necessary to generate models with high classification accuracy. Moreover, the authors investigated the importance of subsets of the microbial community features for the classification process. The taxa identified as more relevant were in line with those identified by previous studies, and classification performance was as well comparable.

In another study, a total of 300 biomarkers were selected from 13,990 features including clinical information and the matrix of relative gene abundance from 806 microbiomes of Chinese individuals (383 controls, 170 with type 2 diabetes, 130 with rheumatoid arthritis, and 123 with liver cirrhosis). Seven algorithms were used, and logistic regression achieved the highest accuracy. This study showed that gut microbiome biomarkers could distinguish abnormal cases from controls with a high level of specificity. The microbiome biomarkers found, present a promising predictive power for application in disease diagnostics, especially disease screening within a large-scale population (Wu et al., 2018).

Tap et al. (2017) set up a ML procedure to identify a microbial signature to predict the severity of Irritable Bowel Syndrome (IBS) using a LASSO-based logistic regression approach applied to 195 subjects. The performance was assessed using the AUROC, and a set of 90 robust OTUs was negatively associated with microbial richness, exhaled methane, presence of methanogens, and enterotypes enriched with the bacterial order *Clostridiales* or genus *Prevotella* (Tap et al., 2017). Fukui et al. (2020) used a similar LASSO logistic regression-based approach to extract a featured group of bacteria for identifying IBS patients. They then applied Random Forest models on the selected features to perform the classification between 85 IBS patients and from 26 healthy controls, obtaining a sensitivity of >80% and specificity of >90% (Fukui et al., 2020).

##### Linear discriminant analysis (LDA)

Linear Discriminant Analysis (LDA) is a generalization of Fisher’s linear discriminant, a method used in statistics, pattern recognition and machine learning to find a linear combination of features that provides good separation between the classes of objects or events. When applied to microbiome data, this approach finds a linear combination of microbial features in the training data that models the multivariate mean differences between classes (Zhou and Gallins, 2019).

The linear discriminant analysis (LDA) effect size (LEfSe) method proposed by The Huttenhower Lab as part of bioBakery workflows for executing microbial community analyses^3^ was specifically designed for biomarker discovery in metagenomic data (16S rRNA gene and whole-genome shotgun datasets). It performs high-dimensional class comparisons that determine the features: organisms, clades, operational taxonomic units, genes, or functions; most likely explaining differences between classes. It joins standard tests for statistical significance plus additional tests encoding biological consistency and effect relevance. The algorithm first uses the non-parametric factorial Kruskal-Wallis (KW) sum-rank test to detect features with significant differential abundance regarding the class of interest. Then, biological consistency is investigated using a set of pairwise tests among subclasses using the (unpaired) Wilcoxon rank-sum test, finally uses LDA to estimate the effect size of each differentially abundant feature and perform dimension reduction (Segata et al., 2011).

##### k-nearest neighbors (k-NN)

k-NN is based on simple classification rule, assigning the new sample to a class which is in the majority among the k training samples nearest to that point. The algorithm can be used both for classification and regression problems, depending on a type of the outcome variable (discrete or continuous). The neighborhood is defined using a selected distance metric in a multidimensional feature space. Euclidean distance or correlation coefficients are the most regularly used distance metrics. For continuous traits, a weighted average of the k nearest neighbor is used (Zhou and Gallins, 2019).

k-NN has been used to effectively determine the postmortem interval (PMI) using microbial samples from the skin microbiota found in the nasal and ear canals of cadavers. When the microbiota from both sites was considered jointly, the regression was successful, yielding a model that accurately predicts the postmortem interval to within 55 accumulated degree days (ADD), which represents about two days of decomposition at an average temperature of 27.5°C (Johnson et al., 2016).

Hacılar et al. (2018) compared several ML-based techniques to classify fecal samples as healthy or with disease [i.e., Inflammatory Bowel Disease (IBD)]. They used a dataset containing shotgun metagenomic data from 382 individuals (234 healthy and 148 IBD patients). The training set was a profile of gut microbial communities for each sample generated by MetaPhlAn2 (Segata et al., 2012). Several models were trained (RF, Adaboost, k-NN + LogitBoost, Decision tree, Neural network, LogitBoost and Furia) and 10-fold cross-validation was performed to evaluate the performance for each model. Finally, they added a feature selection (i.e., mRMR: minimum redundancy and maximal relevance) step before the training process. With and without feature selection k-NN + LogitBoost performed best with 0.87 and 0.86 accuracy scores, respectively (Hacılar et al., 2018).

##### Naïve Bayes classifiers

Naïve Bayes classifiers are a family of simple probabilistic classifiers based on the application of Bayes’ theorem with strong (naïve) assumptions of statistical independence between the features. In one such study applying NB to microbiome data, Werner et al. (2012) investigated the influence of the training set on the results of the taxonomic classification of 16S rRNA gene sequences generated in microbiome studies. The classification using a naïve Bayes classifier indicated that taxonomic classification accuracy of 16S rRNA gene sequences improves when a Naive Bayes classifier is trained only on a selected region of the target sequences. This result was used for some other classifiers (e.g., in QIIME2) (Werner et al., 2012).

##### Support vector machines (SVM)

SVMs is a machine learning algorithm that aims to learn a decision boundary between the classes, so as to ensure the maximum achievable distance (margin) between the samples closest to the decision boundary. The samples relevant for learning a decision boundary are only those closest to it, called support vectors. When linear separation between classes is not possible in original feature space, the SVM uses the kernel trick to estimate the decision boundary in a higher-dimensional space (Cortes and Vapnik, 1995). SVM can as well be used for regression tasks.

A Sino-European team (Qin et al., 2010) led an early study using WGS data in order to identify dissociative genetic markers from fecal sample sequencing data for IBD and Type II diabetes (T2D). They used a variety of tools to process the raw reads: SOAPdenovo (Li et al., 2010) for assembly; MetaGene (Noguchi et al., 2006) for gene prediction; KEGG (Kanehisa et al., 2004) and eggNOG (Jensen et al., 2007) for functional annotation. They selected 50 marker genes for T2D (using mRMR: minimum redundancy and maximal relevance) out of a gene catalog containing roughly 300,000 genes. They also show that taxonomic abundance data segregates IBD and healthy individuals when performing PCoA.

Cui and Zhang (2013) described an alignment-free supervised classification procedure for the classification of metagenome samples into predefined classes with sequence signatures from shotgun metagenomics sequencing data by using recursive SVM, this approach integrates feature selection and classification steps in one method. They also applied the methodology on a real metagenome dataset to classify IBD and non-IBD samples. The accuracy obtained using the stringent leave-one-out cross-validation (LOOCV) was 88%, additionally permutation experiment were performed to evaluate statistical significance (Cui and Zhang, 2013).

Liu Y. et al. (2011) presented “MetaGUN”^4^ a gene prediction method for identifying genes in metagenomic fragments based on SVM. Initially, input sequences were classified into phylogenetic groups, using a k-mer based sequence binning method. Afterward, for each group, the identification of protein-coding sequences was performed using SVM classifiers. MetaGUN applies universal prediction modules and a novel prediction module to identify protein-coding sequences. Entropy density profiles (EDP) of codon usage, Translation Initiation Side (TIS) scores and Open Reading Frame (ORF) length are employed as discriminative features and used as inputs into the classifiers to distinguish protein-coding sequences from non-coding sequences. In the last stage, TISs are relocated by employing a modified version of MetaTISA. The MetaGUN prediction method was compared with six existing metagenomic gene finders (Liu Y. et al., 2011). The results showed that the performance of MetaGUN is better for 3′ end of genes on longer fragments, and comparable results were obtained with Glimmer-MG on shorter fragments. For 5′ end of genes, with fragments of various lengths, MetaGUN outperformed other tested methods on the overall TISs. When applied on two healthy human gut microbiome samples, MetaGUN was able to find more novel genes than other methods (Liu Y. et al., 2011).

Ning and Beiko (2015) explored a phylogenetic approach in classification of oral microbiota using a ML approach focusing on classification using SVMs. The authors used phylogenetic information as the basis for the proposed custom kernels and as classifier features. Other than using the phylogenetic information (such as taxon and clade abundance), PICRUSt (Langille et al., 2013) that predicts molecular functions from 16S rRNA sequence data was used to generate additional input features. The proposed kernels based on UniFrac measure of community dissimilarity (Lozupone et al., 2011) did not result in improved performance. Even though the combinations of the selected input features were important predictors, they did not result in increased accuracy. The classification was performed on nine oral sites and resulted in a modest 81% prediction accuracy which indicates the challenges of classification of oral microbiota.

Another study, performed by Larsen and Dai (2015), demonstrated that the metabolome derived from the human gut microbiome might be predictive of host dysbiosis. Metagenomic enzyme profiles predicted from 16S rRNA microbiome community structures were used to generate metabolic models. The authors apply SVM to show that emergent property of the microbiome and its aggregate community metabolome of human gut are more predictive of dysbiosis than the microbiome community composition or predicted enzyme function profiles.

##### Artificial neural networks

Artificial neural networks refer to an interconnected feed-forward network of neural units each comprising multiple inputs and a single output, organized in several layers to map a feature vector from the input layer, to the class label at the output layer. The inputs to each neuron are weighted outputs from the neurons from a previous layer, which are summed and non-linearly transformed at its output. The total number of hidden layers and the number of neurons within each hidden layer are specified by the user. All neurons from the input layer are connected to all neurons in the first hidden layer, with weights representing each connection. This process continues until the last hidden layer is connected. The backpropagation algorithm is used to modify the weights in a neural network optimizing for the classification accuracy. For microbiome data, OTUs/ASVs are commonly used at the input layer, with separate neurons for each OTU/ASV.

Lo and Marculescu (2019) describe a neural network platform for the classification of host phenotypes from metagenomic data, using a new data augmentation technique to mitigate the effects of data over-fitting. They tested the proposed framework on eight real datasets including data from HMP (Turnbaugh et al., 2007), and two diseases, i.e., IBD (Gevers et al., 2014), and esophagus diseases (esophagitis, Barrett’s esophagus, esophagal adenocarcinoma; Yang et al., 2015), finding that the new proposed methodology outperforms other models previously used in the literature (Lo and Marculescu, 2019).

#### Deep Learning

Deep learning (DL) is a ML method that assumes using artificial neural networks (ANNs) with deep architectures, i.e., multiple hidden layers, yielding a higher level of abstraction and in general a significant improvement in performance given very large data sets. Another advantage to other ML methods is that DL architectures learn the feature representation given the raw data at its input, thus alleviating the feature engineering step. Currently, DL is thought to be the most advanced ML technique for a variety of applications (Chassagnon et al., 2020).

To classify human epithelial materials highly relevant for forensic investigations, Díez López et al. (2019) applied taxonomy-independent DL methods on skin, saliva, and vaginal microbiome data obtained from the Human Microbiome Project. A total of 1636 validated reference samples from these sites were used to identify most informative sequence positions via correspondence analysis. High-inertia positions were used as input matrix to train 50 DL networks based on a 4-layer ANN. Two sets of samples (110 test and 41 mock casework samples) were deployed to validate the output from the deep learning approach with most of the samples being classified correctly. This approach offers a more accurate and efficient tissue-classification approach compared to human biomarkers, as donor DNA-based methods often lead to cross-identification and low specificity due to overlaps in human cell composition. However, a successful application of DL methods in such a context ideally requires standardized biological and methodological conditions during the generation of training and test data (Díez López et al., 2019).

Another example of using DL approach for analyses of metagenomic data are DeepARG networks which are trained to predict antibiotic resistance genes (ARGs) from metagenomic data (Arango-Argoty et al., 2018). DeepARG consists of two models: DeepARG-LS, which was developed to classify ARGs based on full gene length sequences, and DeepARG-SS, which was developed to identify and classify ARGs from short sequence reads. The initial collection of ARGs was obtained from three major databases: CARD, ARDB, and UNIPROT and 30 ARG categories were used to train the models. To further evaluate and validate performance, the DeepARG-LS model was applied to all the ARG sequences in the MEGARes database (Lakin et al., 2017). Also, the ability of the DeepARG-LS model to predict novel ARGs was tested on a set of 76 metallo-beta-lactamase genes obtained from the study of Berglund et al. (2017). Based on the results the authors conclude that the DeepARG models can be used to get an overview or inference of the kinds of antibiotic resistance in a collection of sequences; however, still the downstream experimental validation is required to confirm whether the sequences truly confer resistance.

Asgari et al. (2019) used deep learning, Random Forest(RF) and SVM, for distinguishing among human body-sites, diagnosis of Crohn’s disease, and predicting the environments from representative 16S gene sequences. Moreover, they also proposed a reference- and alignment-free approach for predicting environments and host phenotypes from 16S rRNA gene sequencing data based on k-mer representations. They described that for large datasets (10K samples per class) using DL provides more accurate predictions. However, when the number of samples is not large enough, RFs performed better on both OTUs and k-mer features. However, for classification over representative sequences as opposed to samples (pool of sequences), the SVM outperformed the RF classifier (Asgari et al., 2019).

Convolutional neural network CNNs are similar to traditional deep neural networks (DNNs), they are made up of layers of neurons that have learnable weights and biases. Each neuron receives some inputs, calculates a dot product, and optionally follows it with a non-linear function (Lopez Pinaya et al., 2020). In 2017, this team (Fioravanti et al., 2018) introduced a phylogenetic CNN that would enable the classification of gut microbiome metagenomic data into healthy or IBD phenotypes, summing up to a total of 6 classification tasks. Those phenotypes included the different subtypes of the disease: Crohn’s disease (CD) and Ulcerative Colitis (UC), as well as the state of the pathology (flare or remission) and the part of the intestine that is affected for CD (ileum or colon). The dataset used for training (Sokol et al., 2017) contained bacterial and fungal community (16S rDNA and ITS) from 38 controls and 222 IBD patients. Pre-processing of the raw data was carried out using QIIME2 (Kuczynski et al., 2012), UCLUST (Edgar, 2010) and RAxML (Stamatakis, 2014), in order to get relative abundance, cluster the taxa and build a phylogenetic tree that will then be input to the CNN. A synthetic dataset was also constructed as deep learning performs better when trained on large datasets. To do so, they generated vectors in the Aitchison simplex that is spanned by the “real” dataset. This improved the performance of the CNN, which tends to overfit when trained only on the initial dataset. They compared the performance of their newly crafted CNN with more traditional learning models (LSVM, RF, Multi Layer Perceptron NN) using the Matthews Correlation Coefficient (MCC) as a metric. Overall, for each of the six tasks, the CNN outperformed the other models.

#### Ensemble Methods

Ensemble methods combine multiple classifiers to obtain a better performance than a single classifier.

##### Random forests (RF)

RFs are an example of ensemble learning, in which a complex model is made by combining many simple models. In this case, simple models are decision trees. RFs use a bootstrap resampling on the given dataset to learn each decision tree using a single boostrap set. The final output of a RF is obtained using a majority voting of the individual decision trees. As these are well-studied methods, they are used as baselines for comparison in many studies (Breiman, 2001). The most widely used ML algorithm, RF classifiers have been frequently used along with Least Absolute Shrinkage and Selection Operator (LASSO) for feature selection, for stratification of patients (Flemer et al., 2017; Yachida et al., 2019) and biomarker finding (Koohi-Moghadam et al., 2019; Thomas et al., 2019; Wirbel et al., 2019) and finding of host-microbial signatures to detect fecal contamination in environmental samples (Roguet et al., 2018).

RF has been used for classification of pediatric patients of Crohn’s disease (CD) according to disease state and treatment response by using the alpha diversity of the samples and the genetic risk score (GRS) of each patient (Douglas et al., 2018). They found higher classification accuracy with 16S rRNA datasets than shotgun metagenomics due to the higher contamination of human DNA in the shotgun metagenomes.

Ross et al. (2017) analyzed the impact of cohabitation on the individual composition of the skin microbiome. For the analysis, the authors used 16S rDNA amplicons of bacteria and archaea from 330 skin samples from 17 skin regions of 10 heterosexual cohabiting couples. Analysis was performed using both statistical and ML methods. Their results showed that the two most important factors that affect the skin microbiome are individuality and body region, which is in line with previous studies. The authors also showed that cohabitation strongly influences skin microbial community diversity. When RF method was applied for skin microbiome classification, accuracy greater than 86% was achieved (Ross et al., 2017).

Ai et al. (2019) took advantage of the continuously decreasing price of whole genome sequencing technology to diagnose colorectal cancer (CRC) based on fecal shotgun sequencing data. They used a dataset consisting of French and Austrian cohorts both containing 156 individuals (312 in total; 124 healthy and 188 CRC and adenoma patients). To preprocess the raw reads and produce the relative abundance of each taxon in the gut, they used the GRAMMy tool (Xia et al., 2011). In order to select taxa that best discriminate a healthy sample from a sample displaying tumor-related dysbiosis, ML techniques were implemented; feature (taxon) selection was carried out using information theory (mutual information) and a RF classifier was trained using a 6-fold cross-validation process. This resulted in the selection of a set of taxa whose abundance was a good indicator of the presence or not of CRC related dysbiosis in the gut (Ai et al., 2019).

Rahman et al. (2017) used metagenomes to identify antibiotic resistance genes in the infant gut microbiome. Their findings were in line with previous work showing that there is an increase of resistance gene levels after antibiotics intake, which is followed by the recovery of the microbial community. The authors also found that, over time, the formula feeding influences the gut resistome. A RF model was used to classify resistomes of formula-fed and breast-fed babies. Using feature importance, the trained model was then used in the selection of resistance genes. Furthermore, ML methods were used to select genes that can predict the change in relative abundance of an organism after the intake of vancomycin and cephalosporin antibiotics. The best results were obtained using the boosted decision trees (Rahman et al., 2017).

Yang et al. (2019) applied a RF classifier for forensic identification based on an individual’s microbial sample using a combination of single-nucleotide polymorphisms (SNPs) in the 16S rRNA gene of *Cutibacterium acnes* and skin microbiome OTU table, achieving 93.3% accuracy. Their work also showed that the genotype of *C. acnes* 16S rRNA gene was more stable over time than that of the skin microbiome profile. The proposed method showed promising results for microbiome-based forensic identification (Yang et al., 2019).

Gupta et al. studied a cohort of patients with CRC from India by using shotgun metagenomics. They identified 20 potential microbial taxonomic markers based on their significant association with the health status, and 33 potential microbial gene markers using Weka and the Boruta R packages. They applied RF with the selected biomarkers and combined with two different cohorts from China and Austria successfully discriminated the Indian CRC from healthy microbiomes with high accuracy (Gupta et al., 2019).

Sze and Schloss (2016) conducted a meta-analysis to detect if specific microbiome-based markers can be associated with obesity. The authors selected ten previously published studies, re-calculated OTU tables with the available 16S rRNA sequencing data, applied RF models trained on each data set and tested them on the remaining data sets to predict the obesity status of the subjects. The authors found weak relationships between richness, evenness, and diversity and obesity status. Moreover, they also showed that most studies lack the power to detect small differences in alpha diversity metrics and phylum-level relative abundances. The analysis demonstrated that the ability to reliably classify individuals as obese only based on the composition of their microbiome was limited. The authors concluded that the involvement of the microbiome in obesity is not apparent based on the taxonomic information provided by 16S rRNA gene sequence data (Sze and Schloss, 2016).

Braun et al. (2019) studied patients with quiescent Celiac Disease (CD) and compared their microbiota with both CD and healthy patients. The RF model was used to prioritize taxa that best distinguish relapses from non-relapses. Top three taxa were used to construct the flare index that was significantly different for flare and no-flare samples. Flare index also significantly correlated with microbial richness and microbial dysbiosis index (Braun et al., 2019).

Fabijanić and Vlahoviček (2016) utilized the translational optimization effect, a property of gene regulation, to distinguish subjects with liver cirrhosis from healthy controls using the RF classifier (Fabijanić and Vlahoviček, 2016). Another study that utilized the RF algorithm on gut microbiome data is described by Hasic and Music; the condition studied was Multiple Sclerosis (MS). The results demonstrate the best accuracy in distinguishing control samples from MS samples when genus-level taxa abundances were used as features. The model learned on one dataset was evaluated on another set of the MS samples coming from people living in another country. The classification accuracy on this test set was comparable to the error on the validation set (Telalovic and Azra, 2020).

##### Multiple decision tress

Travisany et al. (2015) proposed an ensemble method for microbial taxa prediction present in a specific environment as well as their abundances using multiple CARTs (classification and regression tree). The authors first constructed a dataset of genomic fragments by collecting genomes from publicly available databases. They built two predictors, one using a dataset with 98 genera of the gastrointestinal tract available from the Human Microbiome Project, and the other with 17 early studied genera of the gastrointestinal tract. They computed the statistics of k-mer frequencies, GC radio and GC skew for each read for a specific environment-associated dataset. The prediction was then performed by majority vote selection of multiple (*n* = 558) CART trees. The proposed method was evaluated using simulated and public human gut microbiome datasets. Using 17 representative genera, the authors achieved an accuracy of 77% in read assignments (Travisany et al., 2015).

##### Gradient boosting (GB)

A ML method that addresses regression and classification problems by generating a prediction model as an ensemble of weak predictors, mostly decision trees, and then averaging predictions over decision trees of fixed sizes. As with other forms of boosting, the process successively computes weights for the poorly predicted samples.

For the gut microbiome, GB has been applied by Zeevi et al. (2015). Their study included a cohort of 800 overweight or obese non-diabetic individuals, in which the gut microbiome was being profiled (relative abundances of 16S rRNA amplicon-based phyla, metagenome-based species and KEGG modules) along with their nutritional profiles, as well as several blood parameters and anthropometrics to successfully predict the post-meal glucose levels for each individual and each meal. Their ML model was based on a stochastic gradient boosting regression (Friedman, 2001). When using stochastic gradient boosting, at each iteration, a randomly selected subsample is drawn from the training data without replacement, which is then used to fit the model. Zeevi et al. used 80% of their samples and 40% of the features. They did not limit the depth of the three, however, it was required that the leaves have at least 60 instances (i.e., meals, in their case). In total, 4000 iterations were used with a learning rate of 0.002. The authors subsequently validated the output from the trained ML model in an independent cohort of 100 participants. Further, they conducted a blinded randomized controlled dietary intervention in another cohort based on the ML-based predictions, observing similar improvements in the post-meal glucose levels, accompanied by consistent alterations to the gut microbiota (Zeevi et al., 2015).

Faust et al. (2012) employed GB to investigate co-occurrence relationships in 16S rRNA data obtained from the Human Microbiome Project. Generalized boosted linear models were fitted using taxa abundance data from source sites to predict abundances of target taxa within targets sites. The analysis was augmented with the integration of a set of similarity and dissimilarity measures (Pearson and Spearman coefficients for correlation, Bray-Curtis and Kullback-Leibler as dissimilarity measures) to finally create a network of co-occurrence and co-exclusion relationships within the analyzed microbiomes. By putting these tools together, the authors were able to reveal that closer related taxa tend to co-occur in special vicinity or environmentally similar habitats whereas phylogenetically more distant microbes with similar functional aptitudes are more likely to compete. A major difficulty in developing this method was taking into account the compositional character of relative abundance data which could lead to spurious correlations. However, coupling permutations and repeated renormalization contributed to maintaining true correlations. While these observations were made on data from the Human Microbiome Project, the computational methodology can be transferred to other research questions involving marker gene sequencing (Faust et al., 2012).

GB has been applied to analyze a combination of 16S rRNA, host transcriptome, epigenome, genotype and dietary data from colonic biopsies of inflammatory bowel disease patients and healthy controls using XgBoost (Ryan et al., 2020). When microbiota information was combined with diet and host genotype, the disease classifications improved significantly, and even more so when host epigenome and microbiota data were combined.

#### Applications of Several Machine Learning Methods

Le Goallec et al. (2020) proposed a framework for building microbiome-derived indicators of host phenotypes of infant age, sex, breastfeeding status, historical antibiotic usage, country of origin, and delivery type. By leveraging five different types of data and their combinations (host demographics (“baseline” data) and the four microbiome data type: BioCyc pathway relative abundance, Co-Abundance Groups (CAGs) relative abundance, MetaPhlAn2 taxa relative abundance, and gene relative abundance, they compared the prediction performances of 8 machine learning methods: 2 different elastic net (Elastic Net Caret and Elastic Net 2) implementations, 2 random forest (RF Caret and RF2) implementations, 2 gradient boosted machine (GBM Caret and GBM2) implementations, support vector machines (SVM, kernels: linear, polynomial of degree 2 and radial), K-nearest neighbors (KNN) and naive Bayes (NB). In their investigation, they found that non-linear models and particularly the Gradient Boosted Machines (Caret) were the most consistently effective at the classification of sex, breastfeeding status, country of origin. For other phenotypes such as age and prior antibiotic usage, the information encoded in the microbiome seems to be linear, as no significant difference was observed between the elastic nets and the tree-based methods. In these cases, linear methods were a better choice, because of the ease of interpretation. The authors concluded that significant pairwise relationships could be detected between phenotypes and biomarkers (Le Goallec et al., 2020).

A UK based team carried out a study aiming at building a hybrid classifier that would perform several classification tasks [IBD presence (1), subtype (2) and severity(3)] (Wingfield et al., 2016). A publicly available dataset of 16S rRNA containing fecal sequencing data from 37 healthy individuals and 122 IBD patients) was used in order to train the three aforementioned models. For each sample, the sequenced reads were pre-processed into taxonomic and functional profiles using QIIME2 (Kuczynski et al., 2012) and PICRUSt (Langille et al., 2013) respectively. Then, a pipeline of three consecutive classifiers (SVM for stages one and two, multilayer perceptron (MLP) for stage three) was developed and the classifiers were cross-validated. The outcomes of the different classification steps were disease-free, IBD remission and IBD active for stage one. Ulcerative colitis (UC), Crohn’s disease and control for stage two and finally mild, moderate and severe for stage three. The average precision scores for the k-fold cross-validations were rather low, 0.71, 0.65 and 0.61 for stages one, two and three respectively, however the average area under the ROC curves were consistently better (ranging from 0.7 to 0.9).

In another study, a framework entitled Phy-PMRFI (Phylogeny-aware modeling for prediction of metagenomic functions using RF Feature Importance), the authors use ML for microbiome functional properties. They integrated quantitative profiles of taxa (abundance counts of OTUs) and biological information derived from the phylogeny of microbial taxa. This approach helped to select taxa at different taxonomic levels that reck in associating a metagenomic sample with the host environmental phenotypes. It implemented a phylogeny and abundance-aware matrix (PAAM) (Wassan et al., 2018b) that combines phylogeny with the abundance counts of microbial taxa. For Phy-PMRFI, the authors used RF to recognize microbial features that are useful for classifying phenotypic groups and improve metagenomic predictions. Afterward, the informative microbial taxa obtained acted as an input to three commonly used MLclassifiers: (1) SVM, (2) Logistic Regression, and (3) Naive Bayes, intending to identify if phylogenetic relatedness is a good predictor of functional similarity. For this, the authors used three microbiome datasets as cases to demonstrate the utility of the Phy-PMRFI framework in predicting functions of metagenomic data. They concluded that inclusion of the phylogenetic measure potentially maximizes the opportunity of classifying microbiome functions according to naturally inherent properties of taxa (Wassan et al., 2019).

Beck and Foster (2014) applied genetic programming, RF and logistic regression to classify microbial communities into bacterial vaginosis (BV) positive and negative categories. Using the mentioned classification models, most important features of the microbial community used to predict BV were also identified. The classification was applied to two different datasets. The authors obtained an accuracy above 90% for Nugent score and above 80% for the Amsel criteria. Even though different sets of most important features were identified by the tested classifiers, the shared features, in general, agree with the previous research (Beck and Foster, 2014).

In the context of the human gut microbiome, Zhu et al. (2020) proposed a DL ensemble feature selection model, Deep Forest, which is based on the RF method to perform microbiome-wide association studies (MWAS). When tested on three data sets using several classifiers, the proposed method achieved better classification performance than SVMs, k-NNs and convolutional neural networks (CNNs). Performance evaluation of Deep Forest was also evaluated in terms of feature selection. The method achieved better results with the selected reduced feature subset. When the selected features were compared to the existing literature, identified microbial biomarkers have found to have a relationship with the diseases (Zhu et al., 2020).

Statnikov et al. (2013) performed a comprehensive evaluation of 18 ML methods and five feature selection methods to perform body site and subject multicategory classification and diagnosis using microbiome data. The evaluation was performed on eight datasets using constructed OTU tables as input features for the ML methods. Performance of evaluated methods was measured using the proportion of correct classifications and relative classifier information metrics. From the evaluated methods, RF, SVM, kernel ridge regression, and Bayesian logistic regression with Laplace priors were among the best-performing methods with statistically similar levels of classification accuracy (Statnikov et al., 2013).

In work published by Eck et al. (2017) two datasets were analyzed. One distinguished skin from gut microbiome samples and the other IBD patients from healthy individuals. Several ML algorithms were applied: Linear SVM, RF, nearest shrunken centroids, logistic regression with l_2_ regularization. The authors measured the most important taxa on species level (applying intergenic spacer profiling of 16S-23S rRNA) for the classification when applying different algorithms. The identification of such taxa facilitates biologically meaningful interpretation of the microbiota-based predictions (Eck et al., 2017).

Hollister et al. (2019) evaluated the relationships of pediatric IBS and abdominal pain with intestinal microbes and fecal metabolites. By leveraging both metagenomic and metabolomic information, and using LASSO feature selection, RF models, and SVM, the authors selected ten features including abundances and distributions of the metabolites, bacterial species, and functional pathways. Features selected were capable of distinguishing pediatric IBS cases from controls with an AUC of 0.93 and ≥ 80% accuracy. Moreover, the bacterial features and metabolites described appeared to be closely linked with abdominal pain and emphasized the importance of the microbiome-gut-brain axis to human health (Hollister et al., 2019).

Pasolli et al. (2016) used the SVM, RF classifiers, LASSO and elastic net regularized multiple logistic regression, Neural Networks and Bayesian logistic regression, and assessed the prediction power of metagenomic data in linking the gut microbiome with disease states (Pasolli et al., 2016).

Liu Z. et al. (2011) developed a method called MetaDistance that integrates SVM and k-NN for multiclass classification and additionally performs feature selection. The proposed method showed good classification accuracy for classifying body sites and skin sites according to 16S rRNA gene data. Besides, the method was demonstrated to be robust for small sample sizes and unbalanced classes (Liu Z. et al., 2011).

Mohammed and Guda (2015) used a consensus-based ensemble of k-NN, SVM, RF, decision stump and Naive Bayes classifier to hierarchically predict enzymes encoded by the human gut microbiome. They further applied their method to analyze the enzyme profiles of lean vs obese and IBD vs non-IBD subjects (Mohammed and Guda, 2015).

Chen et al. (2016) explored the differences between the gut microbiome from three different races (Asian, European and American races), by analyzing the expression levels of their gut microbiome genes. They applied minimum redundancy maximum relevance incremental feature selection methods and four ML methods to determine the most relevant gut microbiome genes that are differentially expressed in individuals from different races. The approaches used were: RF, k-NN, sequential minimal optimization (a type of SVM method where training is performed using the sequential minimal optimization algorithm proposed by Platt (1998), and dagging (a type of meta classifier, where multiple models are built and integrated using majority voting). For performance evaluation, the authors used the overall prediction accuracy and Matthews’s correlation coefficient (MCC). MCC was used since it is a suitable performance measure to evaluate model performance even in the case of imbalanced classes (Chicco and Jurman, 2020). Sequential minimal optimization method achieved the best performance results (overall prediction accuracy 99.6%, MCC 99.3%) in identifying 454 most important differentially expressed genes. The obtained results also show that the first 25 out of the 454 identified genes were observed to achieve accuracy greater than 96% and were analyzed in more detail. The identified genes reflected differences among analyzed races such as eating habits, living environments/geographic localization and metabolic levels, which are also known to influence the gut microbiome (Chen et al., 2016).

In more recent work, Zhou and Gallins (2019) evaluated the most commonly used supervised ML methods for microbiome host trait prediction: regression methods, linear discriminant analysis, SVM, similarity matrices and related kernel methods, k-NN, RFs, gradient boosting for decision trees, and neural networks. The authors first performed a comparative analysis based on the literature review of published work, focusing on 17 reported datasets generated from OTU tables. Additionally, the authors performed their own comparative analysis of the mentioned ML methods using three datasets available from MicrobiomeHD database^5^ (Duvallet et al., 2017). For feature extraction, the authors applied a hierarchical feature engineering (HFE) (Oudah and Henschel, 2018). Among the compared methods, decision tree-based methods, in general, performed well, achieving similar results with the neural network models in the analyzed published literature. Furthermore, by applying HFE for OTU table feature reduction, better performance results were achieved for almost all of the evaluated methods (Zhou and Gallins, 2019).

#### Unsupervised Learning Methods

Unsupervised methods identify apparent patterns in the data, without the use of predefined labels. These are important exploratory tools to examine the data and to determine important data structures and correlation patterns (Zhou and Gallins, 2019).

##### Clustering

Hierarchical clustering is a classic unsupervised learning technique, which builds a hierarchy of nested clusters using a dendrogram, merging or splitting clusters based on different metrics (Zhou and Gallins, 2019). Cai and Sun (2011) used hierarchical clustering for classification of 16S rDNA sequences, they developed ESPRIT-Tree, a hierarchical clustering-based algorithm and demonstrated its utility by performing analysis of millions of 16S rRNA sequences, simultaneously addressing the space and computational issues. The novel algorithm exhibits a quasilinear time and space complexity comparable to greedy heuristic clustering algorithms while achieving a similar accuracy to the standard hierarchical clustering algorithm using 16S rRNA data (Cai and Sun, 2011). In another study, the authors applied hierarchical clustering for establishing possible relations between microbiota and disease-associated host changes, i.e., disease prediction. Here, the authors used as feature transcriptome (RNA-seq) signatures of the host cell (colonocytes), and the 16S rRNA data from gut microbiota. The authors treated colonic epithelial cells with live microbiota from five healthy individuals. Their results show an important role of gut microbiota in regulating host gene expression and suggest that manipulation of microbiome composition could be useful in future therapies (Richards et al., 2019).

Possible correlation between microbiota and disease-associated host changes is done through another microbiome communities clustering algorithm - a novel multivariate testing method called an adaptive Microbiome-based Sum of Powered score (aMiSPU) (Wu et al., 2016). The aMiSPU method is proposed to assess how the compositions of microbiotas are associated with human overall health. Since it is a data-driven approach based on a sum of powered score (SPU) tests and adaptive variable weighting, using a generalized taxon proportion combining microbial abundance information with phylogenetic tree information, it reduces the criticality of the choice of a phylogenetic distance which was a weak point in most previous methods. Most univariate tests depend on strong parametric assumptions on the distributions or mean-variance functional forms for microbiome data which results in a false positive (type I errors). So, some findings are considered significant when they have occurred by chance. As no assumption is imposed, the proposed method - a multivariate semi-parametric test - eliminates the chance of incorrectly rejecting a true null hypothesis that there is no association between any taxa and the outcome of interest. The evaluation of aMiSPU test on simulated and real data indicates that the aMiSPU test is better performing than several competing with well-controlled type I error rates. A by-product of the method is a ranking of the importance of the taxa and be used as a selection tool for the taxa which are likely to be associated with the outcome of interest. The MiSPU R package is public and accessible at https://github.com/ChongWu-Biostat/MiSPU. Its application for understanding the association between microbial communities (i.e., microbiotas) throughout the human body and disease can help in developing personalized medicine.

Biclustering is a powerful data mining technique that allows simultaneously clustering rows and columns of a data matrix to find submatrices that can overlap (Xie et al., 2019). In principle, there exist four categories of biclustering methods: (1) variance minimization methods, (2) two-way clustering methods, (3) motif and pattern recognition methods and (4) probabilistic and generative approaches (Madeira and Oliveira, 2004). For many years, biclustering algorithms have been widely used for the analysis of gene expression data, but new biclustering applications are emerging, such as detecting disease marker genera from gut microbiome as those methods are suitable to detect overlapping clusters on both microbes and hosts. Falony et al. (2016) used biclustering to identify sample subsets with specific taxonomic signatures detecting two stable clusters showing that partially overlapped with previously described enterotypes (Falony et al., 2016). Zhou et al. (2020) proposed an identifiable Bayesian multinomial matrix factorization model to infer overlapping clusters on both microbes and hosts. The authors demonstrate the utility of the proposed approach by comparing four alternative methods in simulations and then by applying it into Qin’s IBD microbiome dataset revealing clusters which contain bacteria families that are known to be related to the inflammatory bowel disease and its subtypes according to biological literature (Zhou et al., 2020).

To cluster groups of communities with similar compositions into envirotypes or enterotypes and thus into “metacommunities” the Dirichlet multinomial mixture (DMM) generative modeling framework has been developed (Holmes et al., 2012). It assesses the community structure, including the sample density and size. Multinomial sampling coupled with Dirichlet prior was used before, but the extension of the prior to a mixture of Dirichlet components is a novelty in this work. The method describes each community by a vector, generated by one of finite possible Dirichlet mixture components with different hyperparameters, where each entry is the probability that a read is from given taxa. These vectors of the frequency of taxa occurrences in each sample are placed in a matrix, which is sparse as most species are observed with low abundance. This multinomial sampling is a discrete model that can be used for assessing the size and sparsity of a community. Moreover, it becomes a starting point for a generative modeling framework which explicitly describes a model for generating the studied data, and provides a means to cluster groups of communities with similar compositions. The product of the research is a software package for fitting DMM models which uses a Laplace approximation to integrate out the hyperparameters and estimate the evidence of the complete model. The authors leveraged the methodology to estimate the association of obesity with distinct microbiota by applying the DMM model to human gut microbe genera frequencies from Obese and Lean twins. They did not find a significant impact of body mass on community structure, but rather a possible relation to a disturbed enterotype. They conclude that disturbed states are associated with a more variable community, as this was observed apart from the obese twins, also in people suffering from inflammatory bowel disease (IBD) and ileal Crohn’s disease (ICD).

##### Non-negative matrix factorization (NMF)

This method aims to extract hidden patterns from a series of high-dimensional vectors automatically and has been widely applied in many areas, such as image and natural language processing, and computational biology for dimensional reduction, unsupervised learning (clustering, semi-supervised clustering and co-clustering, etc.) and prediction (Zhang, 2012). The NMF analysis can provide a range of interpretable conclusions about the data sets. For metagenomic data, the features extracted can be mapped to metabolic pathways.

In the work by Cai et al. (2017), the authors use non-negative matrix factorization to identify key features of microbial communities, by analyzing 16S rDNA amplicon and functional data. Using three data sets: the difference in macrolide synthesis pathways for the non-ruminant herbivores; the change in gut and tongue microbial composition for person two in the moving picture data (Caporaso et al., 2011); and the differences in various pathways for the IBD microbiome dataset (Qin et al., 2010) the authors demonstrate how to interpret the features identified by NMF to draw meaningful biological conclusions and discover hitherto unidentified patterns in the data (Cai et al., 2017).

#### Other ML Methods

##### Causal inference methods

Causal inference methods provide exploratory data analysis of causal relationships between variables, e.g., relationship between microbial species and disease outcome.

###### Bayesian networks (BN)

BN are probabilistic graphical models consisting of a directed acyclic graph (DAG). In this model, nodes correspond to random variables, and the directed edges correspond to potential conditional dependencies between them. In a recent study, authors constructed a BN model via Augmented Markov Blanket algorithm to identify microbial networks and species-related with the complete response after concurrent chemoradiation in rectal cancer. The BN analysis revealed a link between a specific taxon and an improved therapeutic response (Jang et al., 2020). BN has also been used in combination with other methods, in particular, the Intervention calculus when the DAG is absent (IDA) method (Kharrat et al., 2019), to identify microbial species that are likely to have a causal role in colorectal cancer (CRC) risk and onset.

###### Dynamic Bayesian networks (DBNs)

Dynamic Bayesian Networks (DBNs) are BNs attested for modeling relationships over temporal data. In this regard, a DBN is a directed acyclic graph where, at each time slice or instance, nodes correspond to random variables of interest and directed edges correspond to their conditional dependencies in the graph (Russell and Norvig, 2016). DNB has been used for analyzing longitudinal microbiome data sets to establish temporal relationships between different taxonomic ranks and other clinical factors that affect the microbiome (Lugo-Martinez et al., 2019). They studied longitudinal data sets from three human microbiome body sites: infant gut, vagina, and oral cavity, and use temporal alignments to normalize the differences in the progress of biological processes of each subject, they found that microbiome alignments improve the predictive performance of the methodology over previous studies of longitudinal datasets, and increase the ability to infer new and previously reported biological and environmental relationships between the components of the microbiome and other factors that influence it, this methodology allows to predict microbiome states and relationships based on longitudinal data applying DBN. Moreover, authors build up the CGBayesNets package that is freely available under the MIT Open Source license agreement.

In general, time series analyses represent a valuable approach to determine the resilience and variability of microbial communities. Perturbations and changing environmental conditions can drive communities into alternative stable states, while bi- and multi-stable states are mostly induced by member interactions within a microbial community. However, a detailed exploration of these temporal shifts is often restricted by either intensively sampled but small treatment groups or large studies, including only few sampling time points. Faust et al. (2015) compared twelve-time series analysis techniques used for high-throughput sequencing studies. These techniques mostly operate on cross-correlation, autocorrelation or network inference. Although the sampling scheme is highly dependent on the environment of interest, appropriate sampling frequency and regularity are crucial. These parameters define the resolution, completeness, sparsity, and noisiness of the data and potentially limit the explanatory power of the analysis output. By applying DBN techniques, incomplete data may be amended and used to model dependencies in time series. Apart from that, the identification of early warning signs indicating an upcoming change in microbiome-inherent networks could help to predict responses to environmental factors (Faust et al., 2015).

###### Mendelian randomization (MR)

Mendelian randomization (MR) has been used to understand the causal role of gut microbiome in disease. MR uses human genetic variants, such as single nucleotide polymorphisms (SNP), as proxy measures for clinically relevant traits of interest (e.g., gut microbiome) to estimate the causal relationship between a trait and a disease or health outcome, therefore eliminating confounding and reverse causation effects between the exposure of interest and outcome. In a bidirectional MR analysis on over 3800 individuals from the Flemish Gut Flora Project and two German cohorts, Hughes and co-workers (Hughes et al., 2020) were able to estimate relationships among five microbial traits and seven outcomes, namely waist circumference and body mass index.

Also, Sanna et al. (2019) used bidirectional MR to assess the causal role of the gut microbiome on metabolic traits, based on genome-wide genetic information, gut metagenomic sequence and fecal short-chain fatty acid (SCFA) levels from 952 normoglycemic individuals, combined with genome-wide-association summary statistics for 17 metabolic and anthropometric traits. The authors found a causal role of gut-produced fecal SCFA with respect to energy balance and glucose homeostasis. In particular, a genetically influenced shift in the gut microbiome toward increased production of butyrate with beneficial effects on beta-cell function, and host genetic variation resulting in increased fecal propionate levels affecting type 2 diabetes risk (Sanna et al., 2019).

###### Correlation-based network analysis

Seo et al. (2017) studied which of the gut microbes responded to probiotic intervention, and their association with gastrointestinal symptoms in healthy adult humans. The study consisted of 21 individuals after probiotics consumption for 60 days and evaluated the changes in microbiome composition through 16S rRNA amplicon sequencing. They used correlation-based network analysis and dimensionality reduction to assess the effect of probiotics consumption and found that probiotic intervention reduced the abundance of potential bacteria such as *Citrobacter* and *Klebsiella* spp. in the human gut microbial community. Moreover, they found that probiotic intervention may reduce the flatulence through downregulation of *Methanobrevibacter* spp. abundance (Seo et al., 2017).

### Biomedical Applications of ML Techniques in Human Microbiome Analyses

**FIGURE 2:**
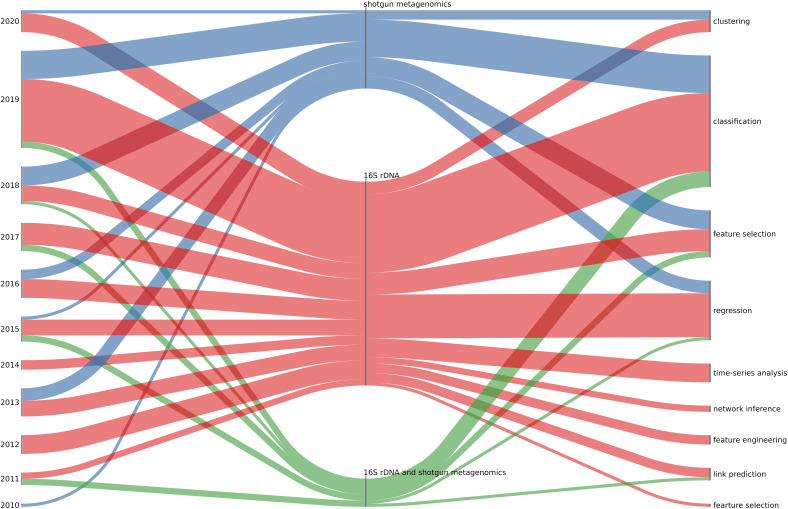
Plot summarizing reviewed articles that apply machine learning in human microbiome data analysis. Articles are summarized based on microbiome input data type and broadly defined ML categories and constrained by year. Please note that in the case of the year 2020 the input does not cover all publications from this year.

Figure 2 summarizes reviewed papers based on the input data type and ML method type. The most dominant input data type in the case application of ML methods for human microbiome analysis has been 16S rRNA amplicon-based sequencing data either in the form of OTU or ASV tables while usage of shotgun metagenomes has increased during recent years. There are a small number of studies that have tested ML methods on both amplicon-based and shotgun datasets. Most often applied ML methods have been feature classification, selection and regression. Most often different ensemble learning methods have been applied while deep learning has been used in few cases. The number of yearly published papers using ML for microbiome data analysis has been slightly growing during years 2011–2018 and increased more than twice in 2019 compared to the previous year (Supplementary Figure 2).

The application of DP to human microbiome analysis is not well captured by our dataset as its application for human microbiome analysis is an emerging field. Recent example includes disease state prediction (inflammatory bowel disease, type 2 diabetes, liver cirrhosis, obesity) using deep representation learning framework that deploys various autoencoders to learn robust low-dimensional representations from high-dimensional microbiome profiles and trains classification models based on the learned representation (Oh and Zhang, 2020) or that relate key microbial biomarkers with metabolite biomarkers in gut microbiome (Le et al., 2020).

**FIGURE 3:**
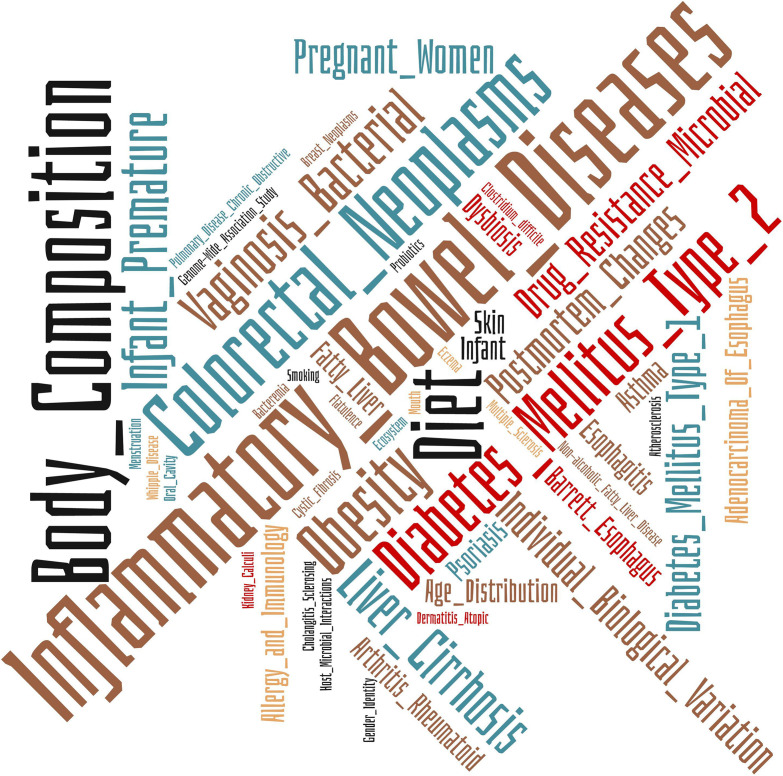
Plot based on Wordcloud with MESH (Medical Subject Headings) terms annotated from the 89 articles.

Our results indicate that the biomedical application of ML for analyses of human microbiome datasets has been mainly focused on the characterization of differently abundant microbial groups between different body sites and the effect of diet on microbiome composition and dynamics. The gut microbiome datasets have been extensively used to stratify and classify patients according to symptoms or characteristics to assist in the diagnosis and management of diseases with a preference on those related with gut microbiome, due to easy accessibility for obtaining fecal samples, such as inflammatory bowel diseases, obesity and colorectal neoplasms (see Figure 3). A list of selected studies on the application of machine learning to human microbiome data in biomedical research is presented in Table 2.

**TABLE 2:** Clinical Applications of Machine Learning for human microbiome studies.

Disease	Datasets	Features	Aim	Method	Citation
Crohn’s Disease (CD)	BISCUIT cohort (Hansen et al., 2012; Pascal et al., 2017), CD *n* = 20, Controls *n* = 20, Validation Cohort RISK cohort (Gevers et al., 2014).	Shotgun metagenomics data and 16S rRNA gene data.	Classify pediatric CD patients by disease state and treatment response.	Random forest.	Douglas et al., 2018
Colorectal Cancer (CRC)	Patients with CRC S0 *n* = 27, Patients with CRC SIII/IV *n* = 54. Healthycontrols *n* = 127.	Shotgun metagenomics data (Species, KO genes, Metabolite profiles).	Classification of CRC patients according to cancer stage.	Feature selection by LASSO. Random forest.	Yachida et al., 2019
Colorectal Cancer (CRC)	Fecal CRC metagenomes: *n* = 38 previously published, *n* = 22 new. Control *n* = 60.	Feature selection by LASSO. Features: IGC gene abundances.	Predict taxonomic and functional microbiome CRC signatures.	Feature selection by LASSO.: Random forest.	Wirbel et al., 2019
Colorectal cancer (CRC)	Stool: Controls *n* = 62, CRC *n* = 69, Polyps *n* = 23. Swabs: Controls *n* = 25, CRC *n* = 45, Polyps *n* = 21.	Log-ratio transformed values of OTUs present in at least 5% of individuals.	Development of an oral and fecal microbiota classifier that distinguish individuals with CRC and adenomas from controls.	Feature selection by LASSO. Random forest.	Flemer et al., 2017
Colorectal cancer (CRC)	Cohort 1: CRC *n* = 29, adenomas *n* = 27, controls = 24. Cohort 2: CRC *n* = 32, Control = 28. Validation Datasets: CRC *n* = 313, Adenomas *n* = 143, Controls = 308.	Taxonomic species-level abundances, gene-family and pathways related abundances.	Finding of reproducible microbiome markers and disease-predictive models for CRC.	Supervised Learning Methods: Random forest.	Thomas et al., 2019
Colorectal cancer (CRC)	Previously published data from France, Hong Kong and Austria. France (Zeller et al., 2014).	Shotgun metagenomics, FASTA.	Discovery of biomarkers from WGS that could be used to build a machine learning classifier for CRC prediction.	Supervised Learning Methods: Random forest. Neural network. Support vector machine.	Koohi-Moghadam et al., 2019
Abnormal cases vs. Controls	Controls *n* = 383. Abnormal Cases: Type 2 diabetes *n* = 170, Rheumatoid arthritis *n* = 130, Liver cirrhosis *n* = 123.	Shotgun metagenomics.	Develop a pipeline to address the challenging characterization of multilabel samples from type 2 diabetes, rheumatoid arthritis, and liver cirrhosis.	Logistic Regression.	Wu et al., 2018)
Bacterial Vaginosis (BV)	Dataset 1: Asymptomatic BV-:299. Asymptomatic BV + :97 Dataset 2: Asymptomatic BV-:6. Asymptomatic BV + :214.	OTU tables from 16S rRNA gene data.	Establishing microbial signatures in bacterial vaginosis (BV).	Logistic Regression, Genetic Programming, and Random Forest.	Beck and Foster, 2015
Colorectal cancer (CRC)	*n* = 30 Controls *n* = 30 CRC patients from Previously published datasets from Austria (*n* = 57 health ycontrols, *n* = 46 CRC patients) and China (*n* = 53 healthy controls and 75 CRC patients) (Feng et al., 2015; Purcell et al., 2017).	Shotgun Metagenomics data (mOTU, MGS, Methaphlan species) Gene counts.	Identify cohort-specific non-invasive biomarkers to be used in diagnosis of CRC.	Weka “CfsSubsetEval” + Boruta algorithm for feature selection. RF with 33 genes and 20 taxonomic markers.	Gupta et al., 2019
Obesity	Data from 10 previously published studies (*n* = 2.786 subjects) (Turnbaugh et al., 2006; Wu et al., 2011; Human Microbiome Project Consortium, 2012; Zupancic et al., 2012; Escobar et al., 2014; Goodrich et al., 2014; Schubert et al., 2014; Ross et al., 2015; Zeevi et al., 2015; Baxter et al., 2016).	OTU tables from 16S rRNA gene data.	Predict obesity status on the basis of the microbial composition of the microbiome.	Random Forest.	Sze and Schloss, 2016
Pediatric irritable bowel syndrome (IBS)	*n* = 23 IBS patients *n* = 22 Healthy Controls.	Shotgun metagenomics, Gene Counts and pathways, Metabolomics.	Evaluate the relationship between pediatric IBS and abdominal pain with intestinal microbes and fecal metabolites.	RF LASSO feature selection SVM naïve Bayes.	Hollister et al., 2019
Gastrointestinal symptoms in healthy humans	*n* = 21 volunteers after probiotics consumption for 60 days.	16S rRNA gene data.	Establish which of the gut microbes respond to probiotics interventions.	Correlation-based network analysis. Dimensionality reduction.	Seo et al., 2017
Chron’s Disease	Chron’s Disease dataset: *n* = 731 Pediatric patients with CD *n* = 628 Non-CD. *n* = 300 healthy controls from HMP (Turnbaugh et al., 2007).	16S rRNA gene data.	Use of deep learning methods and classic machine learning approaches for distinguishing among human body sites, diagnosis of Crohn’s disease, and predicting the environments from representative 16S gene sequences.	RF, SVM, Deep Learning.	Asgari et al., 2019
Inflammatory Bowel Disease (IBD) and esophagus diseases	*n* = 3501 samples from different datasets (Costello et al., 2009; Knights et al., 2011).	16S rRNA gene data.	Classification of metagenomic data using Neural Networks approaches.	Neural Networks. Comparison with supervised ML methods (Linear regression, Boosting gradients, SVM, RF).	Lo and Marculescu, 2019
Islet autoimmunity (IA) and Type 1 Diabetes (T1D).	*n* = 10,913 metagenomes in stool samples from persistent confirmed IA or T1D vs controls. (TEDDY cohort) (Hagopian et al., 2011).	Shotgun metagenomics. Gene count.	Describe the functional profile of the developing gut microbiome in relation to islet autoimmunity, T1D and other early childhood events.	RF to separate between case-controls.	Vatanen et al., 2018
Irritable Bowel Syndrome	71 samples from 22 children with IBS (pediatric Rome III criteria) and 22 healthy children.	16S rRNA gene data.	Finding microbial signatures for Irritable Bowel Syndrome.	Random Forest.	Saulnier et al., 2011
Sclerosing cholangitis	46 controls and 80 patients with PSC during ERC (37 with early disease, 32 with advanced disease, and 11 with biliary dysplasia).	16S rRNA gene data.	Explore the microbial involvement in the etiopathogenesis and risk for development of biliary neoplasia in primary sclerosing cholangitis.	Generalized linear models.	Pereira et al., 2017
Allergy	Skin microbiota samples from 118 individuals.	16S rRNA gene data.	Analyzing atopic sensitization (i.e., allergic disposition) in a random sample of adolescents.	Linear and logistic regression, and PCA.	Hanski et al., 2012
Liver disease	FINRISK population cohort (Borodulin et al., 2018).	Shallow shotgun metagenome sequencing.	Study the link between the Fatty Liver Index (FLI) and gut microbiome composition in a population sample in Finland.	Gradient boosting.	Ruuskanen et al., 2020
Liver disease	A large population-based cohort (*N* ≥ 7,115) and ∼15 years of electronic health register follow-up of the FINRISK population cohort (Borodulin et al., 2018).	Shallow shotgun metagenome sequencing.	Investigate the predictive ability of gut microbial markers in conjunction with conventional risk factors, for incident liver disease and alcoholic liver disease.	Gradient boosting.	Liu et al., 2020
Serum lipids	Healthy Finnish adults (*n* = 25, 18 females, 7 males).	16S rRNA gene data.	Evaluate the association between the gut microbiome and lipid profile.	Linear models, unsupervised hierarchical clustering.	Lahti et al., 2013
IBD (Crohn’s disease, Ulcerative Colitis, collagenous colitis) vs healthy	Three publicly available human metagenomics data sets as Use Cases (Turnbaugh et al., 2009; Koren et al., 2013; Halfvarson et al., 2017).	OTU tables.	Predicting gut microbiome functional role.	Supervised Learning method comparison.	Wassan et al., 2018a
Obesity	267 children aged 7–18 years from the American Gut Project (McDonald et al.).	16S rRNA gene data.	Composition of gut microbiota and its associations with BMI level, weight change and lifestyle.	Linear decomposition model.	Bai et al., 2019
Postmortem Changes	144 sample swabs were from 21 cadavers.	16S rRNA gene data.	Use of necrobiome data in the prediction of the Postmortem interval.	Regression.	Johnson et al., 2016

However, it should be noted that many of the reviewed papers are focused on the comparison of the performance of different ML methods, developing workflows or creating new ML approaches considering the technical aspects of ML related to the nature and complexity of the microbiome data, but without a clear biological or clinical question behind to solve. A detailed analysis of the dataset obtained showed that 20 of 89 papers used their own unique datasets, while the rest of publications made repetitive and intensive use of a limited number of datasets to develop ML solutions, like the Human Microbiome Project widely used for microbiome body composition studies. Besides, we identified 9 papers related to the development of ML methods for microbiome longitudinal analysis that are mainly based on the reuse of five datasets (Caporaso et al., 2011; Gajer et al., 2012; David et al., 2014; La Rosa et al., 2014; DiGiulio et al., 2015) with Gajer et al. being reused in four of them. In addition, we need to highlight the limited sample size in many of the studies what compromises the applicability and the conclusions of the ML methods reviewed.

Table 3 summarizes the main available resources for applying different ML methods to human microbiome studies. Most of the reviewed studies have applied ML methods incorporated in general data analysis packages. As stated by Moreno-Indias et al. (2021). it is important to foster the development of user-friendly ML-based tools for translational and clinical personnel. This process is strongly dependent on open-source software ecosystems as application of ML in microbiome data analysis is rapidly evolving field and involves high degree of multidisciplinary.

**TABLE 3:** Available Resources for applying ML to human microbiome studies.

Tool Name	Description	References
Feature Selection with the R Package MXM	Includes several feature selection algorithms. In particular, the Statistically Equivalent Signatures (SES) algorithm that is very suitable for microbiome data because it scales up to high dimensions and requires few samples. It also reports “multiple biosignatures” meaning multiple, minimal-size subsets of features that lead to an equally predictive model. A more recent feature selection algorithm that scales up well to high dimensional data called Forward-Backward Selection with Early Dropping (FBED) also implemented in the MXM R package; It is preferable to SES when the sample size is higher.	Lagani et al., 2017; Borboudakis and Tsamardinos, 2019
Automated Machine Learning (AutoML) with JADBio.	End-to-end AutoML tool designed to deliver predictive and diagnostic models to non-experts while drastically increasing the productivity of expert analysts. Several qualifications make JADbio (www.jadbio.com) very suitable for microbiome data analysis. First, it accepts numerical measurements (e.g., abundance tables), as well as discrete predictors (e.g. experimental factors and curated metadata), and incomplete datasets with missing values. Second, it facilitates a novel out-of-sample bootstrapping protocol able to provide accurate, non-optimistic estimates of predictive performance even in cases of low sample sizes (e.g., 40) and hundreds of thousands of features Finally. It uses SES and FBED to return the corresponding *biosignatures.* This allows the creation of predictive models that are equally good up to statistical equivalence, thus, providing the researcher with choices when designing new cost-benefit diagnostic assays.	Tsamardinos et al., 2018, 2020
Microbiome network inference with SCENERY.	SCENERY is a free online application that allows users to perform several network learning tasks (scenery.csd.uoc.gr). It is the first of its kind to facilitate advanced algorithms for the inference of association networks, probabilistic causal networks and Bayesian networks. The qualifications of SCENERY have been successfully shown on the single-cell cytometry domain. At the moment, SCENERY does not treat missing values or compositionality, yet, it is readily applicable to the microbiome data domain for inferring causal or non-causal networks of microbiome molecules and species.	Papoutsoglou et al., 2017
The Microbiome Modeling Toolbox	Comprehensive toolbox to model (i) microbe-microbe and host-microbe metabolic interactions, and (ii) microbial communities using microbial genome-scale metabolic reconstructions and metagenomic data.	Baldini et al., 2019
Constraint-based reconstruction and analysis (COBRA) Toolbox v.3.0.	Software suite for quantitative prediction of cellular and multicellular biochemical networks with constraint-based modeling.	Heirendt et al., 2019
Reconstruction, Analysis and Visualization of Metabolic Networks (RAVEN).	RAVEN is a commonly used MATLAB toolbox for genome-scale metabolic model reconstruction, curation and constraint-based modeling and simulation.	Wang et al., 2018
Fizzy: feature subset selection for metagenomics	Python command line tool compatible with BIOM format, for microbial ecologists that implements information-theoretic subset selection methods for biological data formats.	Ditzler et al., 2015; http://github.com/EESI/Fizzy.

Building prediction models for the analysis of microbiome or similar biological data often requires the design of an ML pipeline in which different algorithms for data preprocessing, imputation, feature selection, and modeling are combined along with their hyper-parameter values. The implementation of such a complex modeling strategy could be tedious and requires substantial human resources to optimize. Most importantly, however, this process is prone to serious methodological errors that lead to models whose training performance estimates are inflated (overestimated) and, thus, fail to generalize on external validation datasets. Some common pitfalls of ML application are listed in Table 4.

**TABLE 4:** Common problems in machine-learning analyses.

Problem type	Problem description
Not cross-validating the feature selection step	Perhaps the most common pitfall of performance estimation is that of performing feature selection on the complete, labeled dataset (e.g. by differential expression) and subsequently cross-validating only the modeling algorithm on the same data (Hastie et al., 2009). The same account for any other step on the pipeline that peeks at the labels or the outcome to predict. In the case of large sample and balanced datasets the overestimation should be unnoticeable. On small sample or imbalanced datasets, however, overestimation can become quite significant (Tsamardinos et al., 2020). An analyst should cross-validate all steps of the analysis as atoms, including the preprocessing, imputation, feature selection, and modeling to obtain accurate estimates of performance.
Not correcting for winner’s curse	A second common error is reporting the cross-validation predictive performance of the winning algorithm or ML pipeline as the final performance estimate. For example, an analyst may try 1000 combinations of different algorithms for each step of the analysis with various values for their hyper-parameters and find that the winning combination has a cross-validated accuracy of 80%. This estimate is on average, overestimated because of the “winner’s curse” (Ioannidis, 2008). The overestimation due to the winner’s curse is again large in small or imbalanced datasets. It is not uncommon to find 0.7 AUC when the true one equals random guessing (0.5 AUC) due to the winner’s curse. Other estimation protocols need to be applied in these cases. The simplest solution is to withhold a separate test set to estimate the performance of the winning model; unfortunately, this technique loses samples to estimation and cannot be applied when samples are scarce. Techniques that remove the winner’s curse in small samples are the nested cross-validation and the bootstrap bias-corrected CV (Tsamardinos et al., 2018).
Not stratifying the split to folds	Another typical error occurs when randomly splitting the available samples, either for creating an external validation dataset, or to perform cross-validation, without accounting the class imbalance and sample dependency. The partitioning should be stratified, i.e., the class distribution should be maintained in the folds. When the classes are imbalanced, sample stratification leads to improved performance estimations (Tsamardinos et al., 2015).
Not handling repeated measurements	When sampling is correlated, e.g., the same subject is measured repeatedly, care needs to be exercised. Treating samples as identically and independently distributed (i.i.d.) as cross-validation assumes, provides overestimated performance estimations. When samples are grouped in repeated measurements, one should take care to assign all samples in the group in the same fold. This way, they all belong in the train set or the test set during cross-validation and never in both.
Splitting data inappropriately	When building ML models, typically data is broken into training and test sets. The training set is used to teach the model, and the model’s performance is evaluated by how well it describes the test set. Researchers typically split the data at random that may not be the correct approach always. The “right” way to split data might not be obvious, but careful consideration and trying several approaches may give more insight (Riley, 2019).

## Conclusion

Human microbiome research has received increasing interest during recent years, mainly due to the large potential applicability of metagenomics data from human microbiome studies in personalized medicine. International and interdisciplinary efforts have made possible to collect large volumes of microbiome data, facilitating the development and implementation of different ML methods. Here we reviewed the different ML methods developed and applied to human microbiome data analysis for an insight of the development in the field with their achievements and pitfalls. Although the data presented here is mostly centered on the analysis of bacterial community, many principles reviewed could be applied in general, regardless of the microbiome feature type. The advantages of ML techniques over classical statistical models are to infer relationships between variables for automatic pattern discovery and handling with multi-dimensional data. Therefore, these methods have been widely used for classification, biomarker identification, gene prediction or association studies in human microbiome research. Based on the performed review, most common machine learning algorithms that were used for microbiome analysis were Random Forest, Support Vector Machines, Logistic Regression and k-NN. Since there are several factors that need to be considered during the selection of the ML algorithm (i.e., number of features, number of observations, data quality, data type etc.), it is recommended to apply and evaluate more than one method and select the one with the best performance. However, other ML applications that will be of high interest in the near future are underrepresented like deep learning, spatiotemporal and dynamic modeling, methods for longitudinal and mechanistic analyses or integrative methods for data from different sources to understand microbiome-host interaction and diseases. Nevertheless, the full deployment of ML techniques in human microbiome studies for a complete application and integration in the personalized medicine field requires further efforts. Personalized medicine requires a deep understanding of features characterizing individual particularities and responses a frequent lack of ML methods. ML models with high complexity often come with a loss of interpretability running as black boxes. In many cases, ML methods fail to provide easily, understandable and interpretable predictions essential to identify mistakes or biases in the input data when the model is trained. Moreover, ML methods introduced in this review require fine-tuning of many hyper-parameters to achieve optimal results being a time-consuming task given the high number of possible alternatives. In addition, for training powerful ML methods with reliable results a large amount of data and a lot of computing resources are required. In general, ML methods introduced in this review are based on datasets with a limited number of cases and without other independent datasets what conditions their results and applicability. Therefore, from our review perspective future efforts in the field should be focused in (1) create standards (incl data pre-processing) for the development and deployment of ML techniques with an easy, transparent, and trustable interpretability for non-experts taking in account the peculiarities of microbiome data; (2) increase the number and quality of human microbiome studies; (3) create efficient data structures and ML repositories following Findable, Accessible, Interoperable and Reusable (FAIR) principles and (4) build bridges between different disciplines, microbiology, biology, statistics, bioinformatics, engineering and others to increase interdisciplinary for innovative solutions. COST Action CA18131 on *Statistical and Machine Learning Techniques in Human Microbiome Studies* (ML4Microbiome) is highly committed to pursuit these objectives in collaboration with the international community and extended discussions on contemporary challenges and proposed solutions are addressed by the ML4Microbiome consortium in Moreno-Indias et al. (2021).

## Author Contributions

MC, EC, IM-I, and JT conceived the review. LM-Z and JT coordinated, supervised and wrote the draft, the Supplementary Information and the final manuscript. KK-H, TL, PP, VT, and EC performed the analysis, prepared the figures and Supplementary Information, wrote the draft and the final manuscript. OA, MB, MC, AG, JH, KH, TK, MK, LL, ML, VM, IM-I, IN, EO, IP, GP, RS, BS, BV, EZ, IT, and MY revised draft manuscript, provided comments, included manual references and wrote parts of the final manuscript. All the authors discussed and approved the final version of the manuscript.

## Conflict of Interest

The authors declare that the research was conducted in the absence of any commercial or financial relationships that could be construed as a potential conflict of interest.

## Footnotes

1 https://www.wordclouds.com

2 https://www.microbiomeanalyst.ca/

3 http://huttenhower.sph.harvard.edu/lefse/

4 http://bioinfo.ctb.pku.edu.cn/MetaGUN/

5 https://github.com/cduvallet/microbiomeHD

## References

[B1] Ai D. Pan H. Han R. Li X. Liu G. Xia L. C. (2019). Using decision tree aggregation with random forest model to identify gut microbes associated with colorectal cancer. *Genes* 10:112. 10.3390/genes10020112 30717284 PMC6410271

[B2] Aitchison J. (1986). *The Statistical Analysis of Compositional Data.* London: Chapman and Hall.

[B3] Almeida A. Nayfach S. Boland M. Strozzi F. (2021). A unified catalog of 204,938 reference genomes from the human gut microbiome. *Nat. Biotechnol.* 39 105–114 10.1038/s41587-020-0603-3 32690973 PMC7801254

[B4] Arango-Argoty G. Garner E. Pruden A. Heath L. S. Vikesland P. Zhang L. (2018). DeepARG: a deep learning approach for predicting antibiotic resistance genes from metagenomic data. *Microbiome* 6:23. 10.1186/s40168-018-0401-z PMC5796597 29391044

[B5] Arksey H. O’Malley L. (2005). Scoping studies: towards a methodological framework. *Int. J. Soc. Res. Methodol.* 8 19–32. 10.1080/1364557032000119616

[B6] Asgari E. Garakani K. McHardy A. C. Mofrad M. R. K. (2019). MicroPheno: predicting environments and host phenotypes from 16S rRNA gene sequencing using a k-mer based representation of shallow sub-samples. *Bioinformatics* 35:1082. 10.1093/bioinformatics/bty652 30099528 PMC6419898

[B7] Ashburner M. Ball C. A. Blake J. A. Botstein D. Butler H. Cherry J. M. (2000). Gene ontology: tool for the unification of biology. The gene ontology consortium. *Nat. Genet.* 25 25–29. 10.1038/75556 10802651 PMC3037419

[B8] Bai J. Hu Y. Bruner D. W. (2019). Composition of gut microbiota and its association with body mass index and lifestyle factors in a cohort of 7-18 years old children from the American Gut Project. *Pediatr. Obes.* 14:e12480. 10.1111/ijpo.12480 30417607

[B9] Baldini F. Heinken A. Heirendt L. Magnusdottir S. Fleming R. M. T. Thiele I. (2019). The Microbiome Modeling Toolbox: from microbial interactions to personalized microbial communities. *Bioinformatics* 35 2332–2334. 10.1093/bioinformatics/bty941 30462168 PMC6596895

[B10] Baxter N. T. Ruffin M. T. Rogers M. A. Schloss P. D. (2016). Microbiota-based model improves the sensitivity of fecal immunochemical test for detecting colonic lesions. *Genome Med.* 8:37. 10.1186/s13073-016-0290-3 27056827 PMC4823848

[B11] Beck D. Foster J. A. (2014). Machine learning techniques accurately classify microbial communities by bacterial vaginosis characteristics. *PLoS One* 9:e87830. 10.1371/journal.pone.0087830 24498380 PMC3912131

[B12] Beck D. Foster J. A. (2015). Machine learning classifiers provide insight into the relationship between microbial communities and bacterial vaginosis. *Biodata Min.* 8:23. 10.1186/s13040-015-0055-3 26294933 PMC4542107

[B13] Berglund F. Marathe N. P. Österlund T. Bengtsson-Palme J. Kotsakis S. Flach C.-F. (2017). Identification of 76 novel B1 metallo-β-lactamases through large-scale screening of genomic and metagenomic data. *Microbiome* 5:134. 10.1186/s40168-017-0353-8 29020980 PMC5637372

[B14] Blaxter M. Mann J. Chapman T. Thomas F. Whitton C. Floyd R. (2005). Defining operational taxonomic units using DNA barcode data. *Philos. Trans. R. Soc. Lond. B Biol. Sci.* 360 1935–1943. 10.1098/rstb.2005.1725 16214751 PMC1609233

[B15] Bonder M. J. Abeln S. Zaura E. Brandt B. W. (2012). Comparing clustering and pre-processing in taxonomy analysis. *Bioinformatics* 28 2891–2897. 10.1093/bioinformatics/bts552 22962346

[B16] Borboudakis G. Tsamardinos I. (2019). Forward-backward selection with early dropping. *J. Mach. Learn. Res.* 20 276–314.

[B17] Borodulin K. Tolonen H. Jousilahti P. Jula A. Juolevi A. Koskinen S. (2018). Cohort profile: the national FINRISK STUDY. *Int. J. Epidemiol.* 47 696i–696i. 10.1093/ije/dyx239 29165699

[B18] Braun T. Di Segni A. BenShoshan M. Neuman S. Levhar N. Bubis M. (2019). Individualized dynamics in the gut microbiota precede Crohn’s disease flares. *Am. J. Gastroenterol.* 114 1142–1151. 10.14309/ajg.0000000000000136 30741738

[B19] Breiman L. (2001). Random forests. *Mach. Learn.* 45 5–32. 10.1023/A:1010933404324

[B20] Cai Y. Gu H. Kenney T. (2017). Learning microbial community structures with supervised and unsupervised non-negative matrix factorization. *Microbiome* 5:110. 10.1186/s40168-017-0323-1 28859695 PMC5579944

[B21] Cai Y. Sun Y. (2011). ESPRIT-Tree: hierarchical clustering analysis of millions of 16S rRNA pyrosequences in quasilinear computational time. *Nucleic Acids Res.* 39:e95. 10.1093/nar/gkr349 21596775 PMC3152367

[B22] Callahan B. J. McMurdie P. J. Holmes S. P. (2017). Exact sequence variants should replace operational taxonomic units in marker-gene data analysis. *ISME J.* 11 2639–2643. 10.1038/ismej.2017.119 28731476 PMC5702726

[B23] Caporaso J. G. Lauber C. L. Costello E. K. Berg-Lyons D. Gonzalez A. Stombaugh J. (2011). Moving pictures of the human microbiome. *Genome Biol.* 12:R50. 10.1186/gb-2011-12-5-r50 21624126 PMC3271711

[B24] Chassagnon G. Vakalopolou M. Paragios N. Revel M.-P. (2020). Deep learning: definition and perspectives for thoracic imaging. *Eur. Radiol.* 30 2021–2030. 10.1007/s00330-019-06564-3 31811431

[B25] Chen L. Zhang Y. H. Huang T. Cai Y. D. (2016). Gene expression profiling gut microbiota in different races of humans. *Sci. Rep.* 6:23075. 10.1038/srep23075 26975620 PMC4791684

[B26] Chicco D. Jurman G. (2020). The advantages of the matthews correlation coefficient (MCC) over F1 score and accuracy in binary classification evaluation. *BMC Genom.* 21:6. 10.1186/s12864-019-6413-7 31898477 PMC6941312

[B27] Chong J. Liu P. Zhou G. Xia J. (2020). Using microbiomeanalyst for comprehensive statistical, functional, and meta-analysis of microbiome data. *Nat. Protoc.* 15 799–821. 10.1038/s41596-019-0264-1 31942082

[B28] Cortes C. Vapnik V. (1995). Support-vector networks. *Mach. Learn.* 20 273–297. 10.1007/BF00994018

[B29] Costello E. K. Lauber C. L. Hamady M. Fierer N. Gordon J. I. Knight R. (2009). Bacterial community variation in human body habitats across space and time. *Science* 326 1694–1697. 10.1126/science.1177486 19892944 PMC3602444

[B30] Cui H. Zhang X. (2013). Alignment-free supervised classification of metagenomes by recursive SVM. *BMC Genom.* 14:641. 10.1186/1471-2164-14-641 24053649 PMC3849074

[B31] David L. A. Materna A. C. Friedman J. Campos-Baptista M. I. Blackburn M. C. Perrotta A. (2014). Host lifestyle affects human microbiota on daily timescales. *Genome Biol.* 15:R89. 10.1186/gb-2014-15-7-r89 25146375 PMC4405912

[B32] Díez López C. Vidaki A. Ralf A. Montiel González D. Radjabzadeh D. Kraaij R. (2019). Novel taxonomy-independent deep learning microbiome approach allows for accurate classification of different forensically relevant human epithelial materials. *Forensic Sci. Int. Genet.* 41 72–82. 10.1016/j.fsigen.2019.03.015 31003081

[B33] DiGiulio D. B. Callahan B. J. McMurdie P. J. Costello E. K. Lyell D. J. Robaczewska A. (2015). Temporal and spatial variation of the human microbiota during pregnancy. *Proc. Natl. Acad. Sci. U.S.A.* 112 11060–11065. 10.1073/pnas.1502875112 26283357 PMC4568272

[B34] Ditzler G. Morrison J. C. Lan Y. Rosen G. L. (2015). Fizzy: feature subset selection for metagenomics. *BMC Bioinform.* 16:358. 10.1186/s12859-015-0793-8 26538306 PMC4634798

[B35] Douglas G. M. Hansen R. Jones C. M. A. Dunn K. A. Comeau A. M. Bielawski J. P. (2018). Multi-omics differentially classify disease state and treatment outcome in pediatric Crohn’s disease. *Microbiome* 6:13. 10.1186/s40168-018-0398-3 29335008 PMC5769311

[B36] Duvallet C. Gibbons S. M. Gurry T. Irizarry R. A. Alm E. J. (2017). Meta-analysis of gut microbiome studies identifies disease-specific and shared responses. *Nat. Commun.* 8:1784. 10.1038/s41467-017-01973-8 29209090 PMC5716994

[B37] Eck A. Zintgraf L. M. de Groot E. F. J. de Meij T. G. J. Cohen T. S. Savelkoul P. H. M. (2017). Interpretation of microbiota-based diagnostics by explaining individual classifier decisions. *BMC Bioinform.* 18:441. 10.1186/s12859-017-1843-1 28978318 PMC5628491

[B38] Edgar R. C. (2010). Search and clustering orders of magnitude faster than BLAST. *Bioinformatics* 26 2460–2461. 10.1093/bioinformatics/btq461 20709691

[B39] Elekwachi C. O. Wang Z. Wu X. Rabee A. Forster R. J. (2017). Total rRNA-Seq analysis gives insight into bacterial, fungal, protozoal and archaeal communities in the rumen using an optimized rna isolation method. *Front. Microbiol.* 8:1814. 10.3389/fmicb.2017.01814 28983291 PMC5613150

[B40] Escobar J. S. Klotz B. Valdes B. E. Agudelo G. M. (2014). The gut microbiota of colombians differs from that of Americans, Europeans and Asians. *BMC Microbiol.* 14:311. 10.1186/s12866-014-0311-6 25495462 PMC4275940

[B41] Fabijanić M. Vlahoviček K. (2016). Big data, evolution, and metagenomes: predicting disease from gut microbiota codon usage profiles. *Methods Mol. Biol.* 1415 509–531. 10.1007/978-1-4939-3572-7_26 27115650

[B42] Falony G. Joossens M. Vieira-Silva S. Wang J. Darzi Y. Faust K. (2016). Population-level analysis of gut microbiome variation. *Science* 352 560–564. 10.1126/science.aad3503 27126039

[B43] Faust K. Lahti L. Gonze D. de Vos W. M. Raes J. (2015). Metagenomics meets time series analysis: unraveling microbial community dynamics. *Curr. Opin. Microbiol.* 25 56–66. 10.1016/j.mib.2015.04.004 26005845

[B44] Faust K. Sathirapongsasuti J. F. Izard J. Segata N. Gevers D. Raes J. (2012). Microbial co-occurrence relationships in the human microbiome. *PLoS Comput. Biol.* 8:e1002606. 10.1371/journal.pcbi.1002606 22807668 PMC3395616

[B45] Feng Q. Liang S. Jia H. Stadlmayr A. Tang L. Lan Z. (2015). Gut microbiome development along the colorectal adenoma-carcinoma sequence. *Nat. Commun.* 6:6528. 10.1038/ncomms7528 25758642

[B46] Filzmoser P. Hron K. Templ M. (2018). *Applied Compositional Data Analysis.* Cham: Springer.

[B47] Fioravanti D. Giarratano Y. Maggio V. Agostinelli C. Chierici M. Jurman G. (2018). Phylogenetic convolutional neural networks in metagenomics. *BMC Bioinform.* 19:49. 10.1186/s12859-018-2033-5 29536822 PMC5850953

[B48] Flemer B. Warren R. D. Barrett M. P. Cisek K. Das A. Jeffery I. B. (2017). The oral microbiota in colorectal cancer is distinctive and predictive. *Gut* 67 1454–1463. 10.1136/gutjnl-2017-314814 28988196 PMC6204958

[B49] Franzosa E. A. McIver L. J. Rahnavard G. Thompson L. R. Schirmer M. Weingart G. (2018). Species-level functional profiling of metagenomes and metatranscriptomes. *Nat. Methods* 15 962–968. 10.1038/s41592-018-0176-y 30377376 PMC6235447

[B50] Friedman J. H. (2001). Greedy function approximation: a gradient boosting machine. *Ann. Stat.* 29 1189–1232. 10.1214/aos/1013203451

[B51] Fukui H. Nishida A. Matsuda S. Kira F. Watanabe S. Kuriyama M. (2020). Usefulness of machine learning-based gut microbiome analysis for identifying patients with irritable bowels syndrome. *J. Clin. Med. Res.* 9:403. 10.3390/jcm9082403 32727141 PMC7464323

[B52] Gajer P. Brotman R. M. Bai G. Sakamoto J. Schütte U. M. E. Zhong X. (2012). Temporal dynamics of the human vaginal microbiota. *Sci. Transl. Med.* 4:132ra52. 10.1126/scitranslmed.3003605 22553250 PMC3722878

[B53] Gentleman R. C. Carey V. J. Bates D. M. Bolstad B. Dettling M. Dudoit S. (2004). Bioconductor: open software development for computational biology and bioinformatics. *Genome Biol.* 5:R80. 10.1186/gb-2004-5-10-r80 15461798 PMC545600

[B54] Gevers D. Kugathasan S. Denson L. A. Vázquez-Baeza Y. Van Treuren W. Ren B. (2014). The treatment-naive microbiome in new-onset Crohn’s disease. *Cell Host Microb.* 15 382–392. 10.1016/j.chom.2014.02.005 24629344 PMC4059512

[B55] Gilbert J. A. Blaser M. J. Caporaso J. G. Jansson J. K. Lynch S. V. Knight R. (2018). Current understanding of the human microbiome. *Nat. Med.* 24 392–400. 10.1038/nm.4517 29634682 PMC7043356

[B56] Gloor G. B. Macklaim J. M. Pawlowsky-Glahn V. Egozcue J. J. (2017). Microbiome datasets are compositional: and this is not optional. *Front. Microbiol.* 8:2224. 10.3389/fmicb.2017.02224 29187837 PMC5695134

[B57] Gonzalez A. Navas-Molina J. A. Kosciolek T. McDonald D. Vázquez-Baeza Y. Ackermann G. (2018). Qiita: rapid, web-enabled microbiome meta-analysis. *Nat. Methods* 15 796–798. 10.1038/s41592-018-0141-9 30275573 PMC6235622

[B58] Goodrich J. K. Waters J. L. Poole A. C. Sutter J. L. Koren O. Blekhman R. (2014). Human genetics shape the gut microbiome. *Cell* 159 789–799. 10.1016/j.cell.2014.09.053 25417156 PMC4255478

[B59] Gupta A. Dhakan D. B. Maji A. Saxena R. Vishnu Prasoodanan P. K. Mahajan S. (2019). Association of Flavonifractor plautii, a flavonoid-degrading bacterium, with the gut microbiome of colorectal cancer patients in India. *mSystems* 4:e00438-19. 10.1128/msystems.00438-19 31719139 PMC7407896

[B60] Hacılar H. Nalbantoğlu O. U. Bakir-Güngör B. (2018). “Machine learning analysis of inflammatory bowel disease-associated metagenomics dataset,” in *Proceedings of the 2018 3rd International Conference on Computer Science and Engineering (UBMK)*, Sarajevo.

[B61] Hagopian W. A. Erlich H. Lernmark A. Rewers M. Ziegler A. G. Simell O. (2011). The environmental determinants of diabetes in the young (TEDDY): genetic criteria and international diabetes risk screening of 421 000 infants. *Pediatr. Diabetes* 12 733–743. 10.1111/j.1399-5448.2011.00774.x 21564455 PMC3315186

[B62] Halfvarson J. Brislawn C. J. Lamendella R. Vázquez-Baeza Y. Walters W. A. Bramer L. M. (2017). Dynamics of the human gut microbiome in inflammatory bowel disease. *Nat. Microbiol.* 2:17004. 10.1038/nmicrobiol.2017.4 28191884 PMC5319707

[B63] Hansen R. Russell R. K. Reiff C. Louis P. McIntosh F. Berry S. H. (2012). Microbiota of de-novo pediatric IBD: increased *Faecalibacterium prausnitzii* and reduced bacterial diversity in Crohn’s but not in ulcerative colitis. *Am. J. Gastroenterol.* 107 1913–1922. 10.1038/ajg.2012.335 23044767

[B64] Hanski I. von Hertzen L. Fyhrquist N. Koskinen K. Torppa K. Laatikainen T. (2012). Environmental biodiversity, human microbiota, and allergy are interrelated. *Proc. Natl. Acad. Sci. U.S.A.* 109 8334–8339. 10.1073/pnas.1205624109 22566627 PMC3361383

[B65] Hastie T. Tibshirani R. Friedman J. (2009). *The Elements of Statistical Learning.* New York, NY: Springer, 10.1007/978-0-387-84858-7

[B66] Heirendt L. Arreckx S. Pfau T. Mendoza S. N. Richelle A. Heinken A. (2019). Creation and analysis of biochemical constraint-based models using the COBRA Toolbox v.3.0. *Nat. Protoc.* 14 639–702. 10.1038/s41596-018-0098-2 30787451 PMC6635304

[B67] Hoffman J. I. E. (2019). “Logistic regression,” in *Basic Biostatistics for Medical and Biomedical Practitioners*, ed. Hoffman J. I. E. (Amsterdam: Elsevier), 581–589. 10.1016/b978-0-12-817084-7.00033-4

[B68] Hollister E. B. Oezguen N. Chumpitazi B. P. Luna R. A. Weidler E. M. Rubio-Gonzales M. (2019). Leveraging human microbiome features to diagnose and stratify children with irritable bowel syndrome. *J. Mol. Diagn.* 21 449–461. 10.1016/j.jmoldx.2019.01.006 31005411 PMC6504675

[B69] Holmes I. Harris K. Quince C. (2012). Dirichlet multinomial mixtures: generative models for microbial metagenomics. *PLoS One* 7:e30126. 10.1371/journal.pone.0030126 22319561 PMC3272020

[B70] Hughes D. A. Bacigalupe R. Wang J. Rühlemann M. C. Tito R. Y. Falony G. (2020). Genome-wide associations of human gut microbiome variation and implications for causal inference analyses. *Nat. Microbiol.* 5 1079–1087. 10.1038/s41564-020-0743-8 32572223 PMC7610462

[B71] Human Microbiome Project Consortium (2012). Structure, function and diversity of the healthy human microbiome. *Nature* 486 207–214. 10.1038/nature11234 22699609 PMC3564958

[B72] Ioannidis J. P. A. (2008). Why most discovered true associations are inflated. *Epidemiology* 19 640–648. 10.1097/EDE.0b013e31818131e7 18633328

[B73] Jang B.-S. Chang J. H. Chie E. K. Kim K. Park J. W. Kim M. J. (2020). Gut microbiome composition is associated with a pathologic response after preoperative chemoradiation in patients with rectal cancer. *Int. J. Radiat. Oncol. Biol. Phys.* 107 736–746. 10.1016/j.ijrobp.2020.04.015 32315676

[B74] Jensen L. J. Julien P. Kuhn M. von Mering C. Muller J. Doerks T. (2007). eggNOG: automated construction and annotation of orthologous groups of genes. *Nucleic Acids Res.* 36 D250–D254. 10.1093/nar/gkm796 17942413 PMC2238944

[B75] Jiang P. Green S. J. Chlipala G. E. Turek F. W. Vitaterna M. H. (2019). Reproducible changes in the gut microbiome suggest a shift in microbial and host metabolism during spaceflight. *Microbiome* 7:113. 10.1186/s40168-019-0724-4 31399081 PMC6689164

[B76] Johnson H. R. Trinidad D. D. Guzman S. Khan Z. Parziale J. V. DeBruyn J. M. (2016). A machine learning approach for using the postmortem skin microbiome to estimate the postmortem interval. *PLoS One* 11:e0167370. 10.1371/journal.pone.0167370 28005908 PMC5179130

[B77] Kanehisa M. Goto S. (2000). KEGG: kyoto encyclopedia of genes and genomes. *Nucleic Acids Res.* 28 27–30. 10.1093/nar/28.1.27 10592173 PMC102409

[B78] Kanehisa M. Goto S. Kawashima S. Okuno Y. Hattori M. (2004). The KEGG resource for deciphering the genome. *Nucleic Acids Res.* 32 D277–D280. 10.1093/nar/gkh063 14681412 PMC308797

[B79] Kashyap P. C. Chia N. Nelson H. Segal E. Elinav E. (2017). Microbiome at the frontier of personalized medicine. *Mayo Clin. Proc.* 92 1855–1864. 10.1016/j.mayocp.2017.10.004 29202942 PMC5730337

[B80] Kharrat N. Assidi M. Abu-Elmagd M. Pushparaj P. N. Alkhaldy A. Arfaoui L. (2019). Data mining analysis of human gut microbiota links *Fusobacterium* spp. with colorectal cancer onset. *Bioinformation* 15 372–379. 10.6026/97320630015372 31312073 PMC6614120

[B81] Knights D. Costello E. K. Knight R. (2011). Supervised classification of human microbiota. *FEMS Microbiol. Rev.* 35 343–359. 10.1111/j.1574-6976.2010.00251.x 21039646

[B82] Koohi-Moghadam M. Borad M. J. Tran N. L. Swanson K. R. Boardman L. A. Sun H. (2019). MetaMarker: a pipeline for de novo discovery of novel metagenomic biomarkers. *Bioinformatics* 35 3812–3814. 10.1093/bioinformatics/btz123 30825371 PMC6761932

[B83] Koren O. Knights D. Gonzalez A. Waldron L. Segata N. Knight R. (2013). A guide to enterotypes across the human body: meta-analysis of microbial community structures in human microbiome datasets. *PLoS Comput. Biol.* 9:e1002863. 10.1371/journal.pcbi.1002863 23326225 PMC3542080

[B84] Kuczynski J. Stombaugh J. Walters W. A. González A. Gregory Caporaso J. Knight R. (2012). Using QIIME to Analyze 16S rRNA gene sequences from microbial communities. *Curr. Protoc. Microbiol.* 27 1E.5.1–1E.5.20. 10.1002/9780471729259.mc01e05s27 23184592 PMC4477843

[B85] La Rosa P. S. Warner B. B. Zhou Y. Weinstock G. M. Sodergren E. Hall-Moore C. M. (2014). Patterned progression of bacterial populations in the premature infant gut. *Proc. Natl. Acad. Sci. U.S.A.* 111 12522–12527. 10.1073/pnas.1409497111 25114261 PMC4151715

[B86] Lagani V. Athineou G. Farcomeni A. Tsagris M. Tsamardinos I. (2017). Feature selection with the R Package MXM: discovering statistically equivalent feature subsets. *J. Statist. Softw.* 80 1–25. 10.18637/jss.v080.i07

[B87] Lahti L. Salonen A. Kekkonen R. A. Salojarvi J. Jalanka-Tuovinen J. Palva A. (2013). Associations between the human intestinal microbiota, *Lactobacillus rhamnosus* GG and serum lipids indicated by integrated analysis of high-throughput profiling data. *PeerJ* 1:e32. 10.7717/peerj.32 23638368 PMC3628737

[B88] Lakin S. M. Dean C. Noyes N. R. Dettenwanger A. Ross A. S. Doster E. (2017). MEGARes: an antimicrobial resistance database for high throughput sequencing. *Nucleic Acids Res.* 45 D574–D580. 10.1093/nar/gkw1009 27899569 PMC5210519

[B89] Langille M. G. I. Zaneveld J. Gregory Caporaso J. McDonald D. Knights D. Reyes J. A. (2013). Predictive functional profiling of microbial communities using 16S rRNA marker gene sequences. *Nat. Biotechnol.* 31 814–821. 10.1038/nbt.2676 23975157 PMC3819121

[B90] LaPierre N. Ju C. J.-T. Zhou G. Wang W. (2019). MetaPheno: a critical evaluation of deep learning and machine learning in metagenome-based disease prediction. *Methods* 166 74–82. 10.1016/j.ymeth.2019.03.003 30885720 PMC6708502

[B91] Larsen P. E. Dai Y. (2015). Metabolome of human gut microbiome is predictive of host dysbiosis. *Gigascience* 4:42. 10.1186/s13742-015-0084-3 26380076 PMC4570295

[B92] Le V. Quinn T. P. Tran T. Venkatesh S. (2020). Deep in the bowel: highly interpretable neural encoder-decoder networks predict gut metabolites from gut microbiome. *BMC Genom.* 21:256. 10.1186/s12864-020-6652-7 32689932 PMC7370527

[B93] Le Goallec A. Tierney B. T. Luber J. M. Cofer E. M. Kostic A. D. Patel C. J. (2020). A systematic machine learning and data type comparison yields metagenomic predictors of infant age, sex, breastfeeding, antibiotic usage, country of origin, and delivery type. *PLoS Comput. Biol.* 16:e1007895. 10.1371/journal.pcbi.1007895 32392251 PMC7241849

[B94] Li J. Jia H. Cai X. Zhong H. Feng Q. Sunagawa S. (2014). An integrated catalog of reference genes in the human gut microbiome. *Nat. Biotechnol.* 32 834–841. 10.1038/nbt.2942 24997786

[B95] Li R. Zhu H. Ruan J. Qian W. Fang X. Shi Z. (2010). De novo assembly of human genomes with massively parallel short read sequencing. *Genome Res.* 20 265–272. 10.1101/gr.097261.109 20019144 PMC2813482

[B96] Liu Y. Guo J. Zhu H. (2011). “Gene prediction in metagenomic fragments based on the SVM algorithm,” in *Proceedings of the 2011 4th International Conference on Biomedical Engineering and Informatics (BMEI)*, Shanghai, 10.1109/bmei.2011.6098588

[B97] Liu Z. Hsiao W. Cantarel B. L. Drábek E. F. Fraser-Liggett C. (2011). Sparse distance-based learning for simultaneous multiclass classification and feature selection of metagenomic data. *Bioinformatics* 27 3242–3249. 10.1093/bioinformatics/btr547 21984758 PMC3223360

[B98] Liu Y. Meric G. Havulinna A. S. Teo S. M. Ruuskanen M. Sanders J. (2020). Early prediction of liver disease using conventional risk factors and gut microbiome-augmented gradient boosting. *medRxiv* [Preprint], 10.1101/2020.06.24.20138933 PMC9097589 35354069

[B99] Lo C. Marculescu R. (2019). MetaNN: accurate classification of host phenotypes from metagenomic data using neural networks. *BMC Bioinform.* 20:314. 10.1186/s12859-019-2833-2 31216991 PMC6584521

[B100] Lopez Pinaya W. H. Vieira S. Garcia-Dias R. Mechelli A. (2020). “Convolutional neural networks,” in *Machine Learning*, eds Mechelli A. Vieira S. (Amsterdam: Elsevier), 173–191. 10.1016/b978-0-12-815739-8.00010-9

[B101] Lozupone C. Lladser M. E. Knights D. Stombaugh J. Knight R. (2011). UniFrac: an effective distance metric for microbial community comparison. *ISME J.* 5 169–172. 10.1038/ismej.2010.133 20827291 PMC3105689

[B102] Lugo-Martinez J. Ruiz-Perez D. Narasimhan G. Bar-Joseph Z. (2019). Dynamic interaction network inference from longitudinal microbiome data. *Microbiome* 7:54. 10.1186/s40168-019-0660-3 30940197 PMC6446388

[B103] Madeira S. C. Oliveira A. L. (2004). Biclustering algorithms for biological data analysis: a survey. *IEEE/ACM Trans. Comput. Biol. Bioinform.* 1 24–45. 10.1109/TCBB.2004.2 17048406

[B104] McDonald D. Hyde E. Debelius J. W. Morton J. T. Gonzalez A. Ackermann G. (2018). American gut: an open platform for citizen science microbiome research. *mSystems* 3:e0031-18. 10.1128/mSystems.00031-18 29795809 PMC5954204

[B105] Mitchell A. L. Almeida A. Beracochea M. Boland M. Burgin J. Cochrane G. (2020). MGnify: the microbiome analysis resource in 2020. *Nucleic Acids Res.* 48 D570–D578. 10.1093/nar/gkz1035 31696235 PMC7145632

[B106] Mitchell A. L. Scheremetjew M. Denise H. Potter S. Tarkowska A. Qureshi M. (2018). EBI Metagenomics in 2017: enriching the analysis of microbial communities, from sequence reads to assemblies. *Nucleic Acids Res.* 46 D726–D735. 10.1093/nar/gkx967 29069476 PMC5753268

[B107] Mohammed A. Guda C. (2015). Application of a hierarchical enzyme classification method reveals the role of gut microbiome in human metabolism. *BMC Genomics* 16(Suppl. 7):S16. 10.1186/1471-2164-16-S7-S16 26099921 PMC4474468

[B108] Moher D. Liberati A. Tetzlaff J. Altman D. G., and PRISMA Group (2010). Preferred reporting items for systematic reviews and meta-analyses: the PRISMA statement. *Int. J. Surg.* 8 336–341. 10.1016/j.ijsu.2010.02.007 20171303

[B109] Moher D. Stewart L. Shekelle P. (2015). All in the family: systematic reviews, rapid reviews, scoping reviews, realist reviews, and more. *Syst. Rev.* 4:183. 10.1186/s13643-015-0163-7 26693720 PMC4688988

[B110] Moreno-Indias I. Lahti L. Nedyalkova M. Elbere I. Roshchupkin G. Adilovic M. (2021). Statistical and machine learning techniques in human microbiome studies: contemporary challenges and solutions. *Front. Microbiol.* 12:635781. 10.3389/fmicb.2021.635781 PMC7937616 33692771

[B111] Nielsen H. B. Almeida M. Juncker A. S. Rasmussen S. Li J. Sunagawa S. (2014). Identification and assembly of genomes and genetic elements in complex metagenomic samples without using reference genomes. *Nat. Biotechnol.* 32 822–828. 10.1038/nbt.2939 24997787

[B112] Ning J. Beiko R. G. (2015). Phylogenetic approaches to microbial community classification. *Microbiome* 3:47. 10.1186/s40168-015-0114-5 26437943 PMC4593236

[B113] Noguchi H. Park J. Takagi T. (2006). MetaGene: prokaryotic gene finding from environmental genome shotgun sequences. *Nucleic Acids Res.* 34 5623–5630. 10.1093/nar/gkl723 17028096 PMC1636498

[B114] Oh M. Zhang L. (2020). DeepMicro: deep representation learning for disease prediction based on microbiome data. *Sci. Rep*. 10:6026. 10.1038/s41598-020-63159-5 32265477 PMC7138789

[B115] Oudah M. Henschel A. (2018). Taxonomy-aware feature engineering for microbiome classification. *BMC Bioinform.* 19:227. 10.1186/s12859-018-2205-3 29907097 PMC6003080

[B116] Papoutsoglou G. Athineou G. Lagani V. Xanthopoulos I. Schmidt A. Éliás S. (2017). SCENERY: a web application for (causal) network reconstruction from cytometry data. *Nucleic Acids Res.* 45 W270–W275. 10.1093/nar/gkx448 28525568 PMC5570263

[B117] Pascal V. Pozuelo M. Borruel N. Casellas F. Campos D. Santiago A. (2017). A microbial signature for Crohn’s disease. *Gut* 66 813–822. 10.1136/gutjnl-2016-313235 28179361 PMC5531220

[B118] Pasolli E. Schiffer L. Manghi P. Renson A. Obenchain V. Truong D. T. (2017). Accessible, curated metagenomic data through ExperimentHub. *Nat. Methods* 14 1023–1024. 10.1038/nmeth.4468 29088129 PMC5862039

[B119] Pasolli E. Truong D. T. Malik F. Waldron L. Segata N. (2016). Machine learning meta-analysis of large metagenomic datasets: tools and biological insights. *PLoS Comput. Biol.* 12:e1004977. 10.1371/journal.pcbi.1004977 27400279 PMC4939962

[B120] Pawlowsky-Glahn V. Egozcue J. J. Tolosana-Delgado R. (2015). *Modeling and Analysis of Compositional Data.* Chichester: John Wiley & Sons.

[B121] Pereira P. Aho V. Arola J. Boyd S. Jokelainen K. Paulin L. (2017). Bile microbiota in primary sclerosing cholangitis: impact on disease progression and development of biliary dysplasia. *PLoS One* 12:e0182924. 10.1371/journal.pone.0182924 28796833 PMC5552186

[B122] Petersen C. Round J. L. (2014). Defining dysbiosis and its influence on host immunity and disease. *Cell. Microbiol.* 16 1024–1033. 10.1111/cmi.12308 24798552 PMC4143175

[B123] Platt J. C. (1998). Sequential Minimal Optimization: A Fast Algorithm for Training Support Vector Machines. Technical Report MSR-TR-98-14, Microsoft Research.

[B124] Plaza Oñate F. Le Chatelier E. Almeida M. Cervino A. C. L. Gauthier F. Magoulès F. (2019). MSPminer: abundance-based reconstitution of microbial pan-genomes from shotgun metagenomic data. *Bioinformatics* 35 1544–1552. 10.1093/bioinformatics/bty830 30252023 PMC6499236

[B125] Purcell R. V. Visnovska M. Biggs P. J. Schmeier S. Frizelle F. A. (2017). Distinct gut microbiome patterns associate with consensus molecular subtypes of colorectal cancer. *Sci. Rep.* 7:11590. 10.1038/s41598-017-11237-6 28912574 PMC5599497

[B126] Qin J. Li R. Raes J. Arumugam M. Burgdorf K. S. Manichanh C. (2010). A human gut microbial gene catalogue established by metagenomic sequencing. *Nature* 464 59–65. 10.1038/nature08821 20203603 PMC3779803

[B127] Quinn T. P. Erb I. (2020). Interpretable log contrasts for the classification of health biomarkers: a new approach to balance selection. *mSystems* 5:e00230-19. 10.1128/mSystems.00230-19 32265314 PMC7141889

[B128] Quinn T. P. Erb I. Richardson M. F. Crowley T. M. (2018). Understanding sequencing data as compositions: an outlook and review. *Bioinformatics* 34 2870–2878. 10.1093/bioinformatics/bty175 29608657 PMC6084572

[B129] Rahman S. F. Olm M. R. Morowitz M. J. Banfield J. F. (2017). Machine learning leveraging genomes from metagenomes identifies influential antibiotic resistance genes in the infant gut microbiome. *bioRxiv* [Preprint], 10.1101/185348 PMC5758725 29359195

[B130] Randolph T. W. Zhao S. Copeland W. Hullar M. Shojaie A. (2018). Kernel-penalized regression for analysis of microbiome data. *Ann. Appl. Stat.* 12 540–566. 10.1214/17-AOAS1102 30224943 PMC6138053

[B131] Richards A. L. Muehlbauer A. L. Alazizi A. Burns M. B. Findley A. Messina F. (2019). Gut microbiota has a widespread and modifiable effect on host gene regulation. *mSystems* 4:e00323-18. 10.1128/mSystems.00323-18 31481602 PMC6722422

[B132] Riley P. (2019). Three pitfalls to avoid in machine learning. *Nature* 572 27–29. 10.1038/d41586-019-02307-y 31363197

[B133] Rivera-Pinto J. Egozcue J. J. Pawlowsky-Glahn V. Paredes R. Noguera-Julian M. Calle M. L. (2018). Balances: a new perspective for microbiome analysis. *mSystems* 3:e0053-18. 10.1128/mSystems.00053-18 30035234 PMC6050633

[B134] Roguet A. Eren A. M. Newton R. J. McLellan S. L. (2018). Fecal source identification using random forest. *Microbiome* 6:185. 10.1186/s40168-018-0568-3 30336775 PMC6194674

[B135] Ross A. A. Doxey A. C. Neufeld J. D. (2017). The skin microbiome of cohabiting couples. *mSystems* 2:e0043-17. 10.1128/mSystems.00043-17 28761935 PMC5527301

[B136] Ross M. C. Muzny D. M. McCormick J. B. Gibbs R. A. Fisher-Hoch S. P. Petrosino J. F. (2015). 16S gut community of the cameron county hispanic cohort. *Microbiome* 3:7. 10.1186/s40168-015-0072-y 25763184 PMC4355967

[B137] Russell S. J. Norvig P. (2016). *Artificial Intelligence: a Modern Approach.* Malaysia: Pearson Education Limited.

[B138] Ruuskanen M. O. Åberg F. Männistö V. Havulinna A. S. Méric G. Liu Y. (2020). Links between gut microbiome composition and fatty liver disease in a large population sample. *medRxiv* [Preprint], 10.1101/2020.07.30.20164962 PMC7928040 33651661

[B139] Ryan F. J. Ahern A. M. Fitzgerald R. S. Laserna-Mendieta E. J. Power E. M. Clooney A. G. (2020). Colonic microbiota is associated with inflammation and host epigenomic alterations in inflammatory bowel disease. *Nat. Commun.* 11:1512. 10.1038/s41467-020-15342-5 32251296 PMC7089947

[B140] Sanna S. van Zuydam N. R. Mahajan A. Kurilshikov A. Vich Vila A. Võsa U. (2019). Causal relationships among the gut microbiome, short-chain fatty acids and metabolic diseases. *Nat. Genet.* 51 600–605. 10.1038/s41588-019-0350-x 30778224 PMC6441384

[B141] Saulnier D. M. Riehle K. Mistretta T.-A. Diaz M.-A. Mandal D. Raza S. (2011). Gastrointestinal microbiome signatures of pediatric patients with irritable bowel syndrome. *Gastroenterology* 141 1782–1791. 10.1053/j.gastro.2011.06.072 21741921 PMC3417828

[B142] Scholz M. Ward D. V. Pasolli E. Tolio T. Zolfo M. Asnicar F. (2016). Strain-level microbial epidemiology and population genomics from shotgun metagenomics. *Nat. Methods* 13 435–438. 10.1038/nmeth.3802 26999001

[B143] Schubert A. M. Rogers M. A. M. Ring C. Mogle J. Petrosino J. P. Young V. B. (2014). Microbiome data distinguish patients with *Clostridium difficile* infection and non-*C. difficile*-associated diarrhea from healthy controls. *mBio* 5:e001021-14. 10.1128/mBio.01021-14 24803517 PMC4010826

[B144] Segata N. Izard J. Waldron L. Gevers D. Miropolsky L. Garrett W. S. (2011). Metagenomic biomarker discovery and explanation. *Genome Biol.* 12:R60. 10.1186/gb-2011-12-6-r60 21702898 PMC3218848

[B145] Segata N. Waldron L. Ballarini A. Narasimhan V. Jousson O. Huttenhower C. (2012). Metagenomic microbial community profiling using unique clade-specific marker genes. *Nat Methods* 9 811–814. 10.1038/nmeth.2066 22688413 PMC3443552

[B146] Seo M. Heo J. Yoon J. Kim S.-Y. Kang Y.-M. Yu J. (2017). *Methanobrevibacter* attenuation via probiotic intervention reduces flatulence in adult human: a non-randomised paired-design clinical trial of efficacy. *PLoS One* 12:e0184547. 10.1371/journal.pone.0184547 28937980 PMC5609747

[B147] Silverman J. D. Washburne A. D. Mukherjee S. David L. A. (2017). A phylogenetic transform enhances analysis of compositional microbiota data. *eLife* 6:e21887. 10.7554/eLife.21887 28198697 PMC5328592

[B148] Sokol H. Leducq V. Aschard H. Pham H.-P. Jegou S. Landman C. (2017). Fungal microbiota dysbiosis in IBD. *Gut* 66 1039–1048. 10.1136/gutjnl-2015-310746 26843508 PMC5532459

[B149] Stamatakis A. (2014). RAxML version 8: a tool for phylogenetic analysis and post-analysis of large phylogenies. *Bioinformatics* 30 1312–1313. 10.1093/bioinformatics/btu033 24451623 PMC3998144

[B150] Statnikov A. Henaff M. Narendra V. Konganti K. Li Z. Yang L. (2013). A comprehensive evaluation of multicategory classification methods for microbiomic data. *Microbiome* 1:11. 10.1186/2049-2618-1-11 24456583 PMC3960509

[B151] Sze M. A. Schloss P. D. (2016). Looking for a signal in the noise: revisiting obesity and the microbiome. *mBio* 7:e01018-16. 10.1128/mBio.01018-16 27555308 PMC4999546

[B152] Tap J. Derrien M. Tornblom H. Brazeilles R. Cools-Portier S. Dore J. (2017). Identification of an intestinal microbiota signature associated with severity of irritable bowel syndrome. *Gastroenterology* 152 111–123.e8. 10.1053/j.gastro.2016.09.049 27725146

[B153] Telalovic H. J. Azra M. (2020). Using data science for medical decision making case: role of gut microbiome in multiple sclerosis. *BMC Med. Inform. Decis. Mak.* 20:262. 10.1186/s12911-020-01263-2 33046051 PMC7549194

[B154] Thomas A. M. Manghi P. Asnicar F. Pasolli E. Armanini F. Zolfo M. (2019). Metagenomic analysis of colorectal cancer datasets identifies cross-cohort microbial diagnostic signatures and a link with choline degradation. *Nat. Med.* 25 667–678. 10.1038/s41591-019-0405-7 30936548 PMC9533319

[B155] Travisany D. Galarce D. Maass A. Assar R. (2015). “predicting the metagenomics content with multiple CART trees,” in *Mathematical Models in Biology: Bringing Mathematics to Life*, eds Zazzu V. Ferraro M. B. Guarracino M. R. (Cham: Springer International Publishing), 145–160. 10.1007/978-3-319-23497-7_11

[B156] Truong D. T. Franzosa E. A. Tickle T. L. Scholz M. Weingart G. Pasolli E. (2015). MetaPhlAn2 for enhanced metagenomic taxonomic profiling. *Nat. Methods* 12 902–903. 10.1038/nmeth.3589 26418763

[B157] Tsamardinos I. Charonyktakis P. Lakiotaki K. Borboudakis G. Zenklusen J. C. Juhl H. (2020). Just add data: automated predictive modeling and biosignature discovery. *bioRxiv* [Preprint], 10.1101/2020.05.04.075747 PMC9203777 35710826

[B158] Tsamardinos I. Greasidou E. Borboudakis G. (2018). Bootstrapping the out-of-sample predictions for efficient and accurate cross-validation. *Mach. Learn.* 107 1895–1922. 10.1007/s10994-018-5714-4 30393425 PMC6191021

[B159] Tsamardinos I. Rakhshani A. Lagani V. (2015). Performance-estimation properties of cross-validationbased protocols with simultaneous hyper-parameter optimization. *Int. J. Artif. Intell. Tools* 24 1–14. 10.1007/978-3-319-07064-3_1

[B160] Turnbaugh P. J. Ley R. E. Hamady M. Fraser-Liggett C. M. Knight R. Gordon J. I. (2007). The human microbiome project. *Nature* 449 804–810. 10.1038/nature06244 17943116 PMC3709439

[B161] Turnbaugh P. J. Ley R. E. Mahowald M. A. Magrini V. Mardis E. R. Gordon J. I. (2006). An obesity-associated gut microbiome with increased capacity for energy harvest. *Nature* 444 1027–1031. 10.1038/nature05414 17183312

[B162] Turnbaugh P. J. Ridaura V. K. Faith J. J. Rey F. E. Knight R. Gordon J. I. (2009). The effect of diet on the human gut microbiome: a metagenomic analysis in humanized gnotobiotic mice. *Sci. Transl. Med.* 1:6ra14. 10.1126/scitranslmed.3000322 20368178 PMC2894525

[B163] Vangay P. Hillmann B. M. Knights D. (2019). Microbiome Learning Repo (ML Repo): a public repository of microbiome regression and classification tasks. *Gigascience* 8:giz042. 10.1093/gigascience/giz042 31042284 PMC6493971

[B164] Vatanen T. Franzosa E. A. Schwager R. Tripathi S. Arthur T. D. Vehik K. (2018). The human gut microbiome in early-onset type 1 diabetes from the TEDDY study. *Nature* 562 589–594. 10.1038/s41586-018-0620-2 30356183 PMC6296767

[B165] Vervier K. Mahé P. Tournoud M. Veyrieras J.-B. Vert J.-P. (2016). Large-scale machine learning for metagenomics sequence classification. *Bioinformatics* 32 1023–1032. 10.1093/bioinformatics/btv683 26589281 PMC4896366

[B166] Wang H. Marcišauskas S. Sánchez B. J. Domenzain I. Hermansson D. Agren R. (2018). RAVEN 2.0: a versatile toolbox for metabolic network reconstruction and a case study on *Streptomyces coelicolor*. *PLoS Comput. Biol.* 14:e1006541. 10.1371/journal.pcbi.1006541 30335785 PMC6207324

[B167] Wassan J. T. Wang H. Browne F. Zheng H. (2018a). A comprehensive study on predicting functional role of metagenomes using machine learning methods. *IEEE/ACM Trans. Comput. Biol. Bioinform.* 16 751–763. 10.1109/TCBB.2018.2858808 30040657

[B168] Wassan J. T. Wang H. Browne F. Zheng H. (2018b). “PAAM-ML: a novel phylogeny and abundance aware machine learning modelling approach for microbiome classification,” in *Proceedings of the 2018 IEEE International Conference on Bioinformatics and Biomedicine (BIBM)*, Madrid, 10.1109/BIBM.2018.8621382

[B169] Wassan J. T. Wang H. Browne F. Zheng H. (2019). Phy-PMRFI: phylogeny-aware prediction of metagenomic functions using random forest feature importance. *IEEE Trans. Nanobiosci.* 18 273–282. 10.1109/tnb.2019.2912824 31021803

[B170] Weiss S. Xu Z. Z. Peddada S. Amir A. Bittinger K. Gonzalez A. (2017). Normalization and microbial differential abundance strategies depend upon data characteristics. *Microbiome* 5:27. 10.1186/s40168-017-0237-y 28253908 PMC5335496

[B171] Wen C. Zheng Z. Shao T. Liu L. Xie Z. Le Chatelier E. (2017). Quantitative metagenomics reveals unique gut microbiome biomarkers in ankylosing spondylitis. *Genome Biol.* 18:142. 10.1186/s13059-017-1271-6 28750650 PMC5530561

[B172] Werner J. J. Koren O. Hugenholtz P. DeSantis T. Z. Walters W. A. Caporaso J. G. (2012). Impact of training sets on classification of high-throughput bacterial 16s rRNA gene surveys. *ISME J.* 6 94–103. 10.1038/ismej.2011.82 21716311 PMC3217155

[B173] Winand R. Bogaerts B. Hoffman S. Lefevre L. Delvoye M. Van Braekel J. (2020). Targeting the 16s rRNA gene for bacterial identification in complex mixed samples: Comparative evaluation of second (Illumina) and third (oxford nanopore technologies) generation sequencing technologies. *Int. J. Mol. Sci.* 21:298. 10.3390/ijms21010298 PMC6982111 31906254

[B174] Wingfield B. Coleman S. McGinnity T. M. Bjourson A. J. (2016). “A metagenomic hybrid classifier for paediatric inflammatory bowel disease,” in *Proceedings of the 2016 International Joint Conference on Neural Networks (IJCNN)*, Vancouver, BC, 10.1109/ijcnn.2016.7727318

[B175] Wirbel J. Pyl P. T. Kartal E. Zych K. Kashani A. Milanese A. (2019). Meta-analysis of fecal metagenomes reveals global microbial signatures that are specific for colorectal cancer. *Nat. Med.* 25 679–689. 10.1038/s41591-019-0406-6 30936547 PMC7984229

[B176] Wu C. Chen J. Kim J. Pan W. (2016). An adaptive association test for microbiome data. *Genome Med.* 8:56. 10.1186/s13073-016-0302-3 27198579 PMC4872356

[B177] Wu G. D. Chen J. Hoffmann C. Bittinger K. Chen Y.-Y. Keilbaugh S. A. (2011). Linking long-term dietary patterns with gut microbial enterotypes. *Science* 334 105–108. 10.1126/science.1208344 21885731 PMC3368382

[B178] Wu H. Cai L. Li D. Wang X. Zhao S. Zou F. (2018). Metagenomics biomarkers selected for prediction of three different diseases in chinese population. *Biomed Res. Int.* 2018:2936257. 10.1155/2018/2936257 29568746 PMC5820663

[B179] Xia L. C. Cram J. A. Chen T. Fuhrman J. A. Sun F. (2011). Accurate genome relative abundance estimation based on shotgun metagenomic reads. *PLoS One* 6:e27992. 10.1371/journal.pone.0027992 22162995 PMC3232206

[B180] Xie J. Ma A. Fennell A. Ma Q. Zhao J. (2019). It is time to apply biclustering: a comprehensive review of biclustering applications in biological and biomedical data. *Brief. Bioinform.* 20 1449–1464. 10.1093/bib/bby014 29490019 PMC6931057

[B181] Yachida S. Mizutani S. Shiroma H. Shiba S. Nakajima T. Sakamoto T. (2019). Metagenomic and metabolomic analyses reveal distinct stage-specific phenotypes of the gut microbiota in colorectal cancer. *Nat. Med.* 25 968–976. 10.1038/s41591-019-0458-7 31171880

[B182] Yang J. Tsukimi T. Yoshikawa M. Suzuki K. Takeda T. Tomita M. (2019). *Cutibacterium acnes* (*Propionibacterium acnes*) 16S rRNA genotyping of microbial samples from possessions contributes to owner identification. *mSystems* 4:e001672-17. 10.1128/mSystems.00594-19 31771975 PMC6880042

[B183] Yang L. Yachimski P. S. Brodie E. Nelson K. E. Pei Z. (2015). “Foregut microbiome, development of esophageal adenocarcinoma, project,” in *Encyclopedia of Metagenomics*, eds. S. K. Highlander, F. Rodriguez-Valera and B A. White (Cham: Springer), 186–189. 10.1007/978-1-4899-7475-4_709

[B184] Yarza P. Yilmaz P. Panzer K. Glöckner F. O. Reich M. (2017). A phylogenetic framework for the kingdom Fungi based on 18S rRNA gene sequences. *Mar. Genom.* 36 33–39. 10.1016/j.margen.2017.05.009 28578827

[B185] Zdravevski E. Lameski P. Trajkovik V. Chorbev I. Goleva R. Pombo N. (2019). “Automation in systematic, scoping and rapid reviews by an NLP toolkit: a case study in enhanced living environments,” in *Enhanced Living Environments. Lecture Notes in Computer Science*, Vol. 11369 eds Ganchev I. Garcia N. Dobre C. Mavromoustakis C. Goleva R. (Cham: Springer), 1–18. 10.1007/978-3-030-10752-9_1

[B186] Zeevi D. Korem T. Zmora N. Israeli D. Rothschild D. Weinberger A. (2015). Personalized nutrition by prediction of glycemic responses. *Cell* 163 1079–1094. 10.1016/j.cell.2015.11.001 26590418

[B187] Zeller G. Tap J. Voigt A. Y. Sunagawa S. Kultima J. R. Costea P. I. (2014). Potential of fecal microbiota for early-stage detection of colorectal cancer. *Mol. Syst. Biol.* 10:766. 10.15252/msb.20145645 25432777 PMC4299606

[B188] Zhang Z.-Y. (2012). “Nonnegative matrix factorization: models, algorithms and applications,” in *Data Mining: Foundations and Intelligent Paradigms: Volume 2: Statistical, Bayesian, Time Series and other Theoretical Aspects*, eds Holmes D. E. Jain L. C. (Berlin: Springer), 99–134. 10.1007/978-3-642-23241-1_6

[B189] Zhou F. He K. Li Q. Chapkin R. S. Ni Y. (2020). Bayesian biclustering for microbial metagenomic sequencing data via multinomial matrix factorization. *arXiv* [Preprint], Available online at: http://arxiv.org/abs/2005.08361 (accessed 08 February, 2021). 10.1093/biostatistics/kxab002 PMC9291645 33634824

[B190] Zhou Y.-H. Gallins P. (2019). A review and tutorial of machine learning methods for microbiome host trait prediction. *Front. Genet.* 10:579. 10.3389/fgene.2019.00579 31293616 PMC6603228

[B191] Zhu Q. Li B. He T. Li G. Jiang X. (2020). Robust biomarker discovery for microbiome-wide association studies. *Methods* 173 44–51. 10.1016/j.ymeth.2019.06.012 31238097

[B192] Zupancic M. L. Cantarel B. L. Liu Z. Drabek E. F. Ryan K. A. Cirimotich S. (2012). Analysis of the gut microbiota in the old order Amish and its relation to the metabolic syndrome. *PLoS One* 7:e43052. 10.1371/journal.pone.0043052 22905200 PMC3419686

