# Supplementary material for: Applications of Machine Learning in Human Microbiome Studies: A Review on Feature Selection, Biomarker Identification, Disease Prediction and Treatment
Source: Front Microbiol. 2021 Feb 19;12:634511. doi: 10.3389/fmicb.2021.634511 (PMC7962872; doi:10.3389/fmicb.2021.634511)
Supplement: Supplementary file 2 [file Data_Sheet_2.docx]

Supplementary Material

# Supplementary Data

## Mining, screening, and organization of relevant publications

This study adopted a scoping review methodology to identify and analyse the literature on state-of-the-art machine learning (ML) methods and the respective software applied in human microbiome studies. Three approaches were used to gather initial literature corpus for this review:

- A manual search as a collaborative effort of the COST Action No. CA18131 members.
- Natural Language Processing (NLP) automated search performed using NLP Toolkit (Zdravevski et al., 2019) based on abstract and title analysis performed on three major digital libraries: IEEE Xplore, Springer, and PubMed.
- A NLP supported automated search through the available GitHub resources to identify relevant software repositories, and extract corresponding scientific papers. Learning to rank approach was further used for an automatic study relevance assessment.

The methods relying on more sophisticated algorithms provide for an efficient and exhaustive search of available research evidence, surpassing the potentials of manual search. In this supplementary section we provide more detailed description of two complementary approaches used for the study collection, screening and eligibility/relevance assessment of studies: the NLP Toolkit based and learning to rank approach.

### **NLP Toolkit supported publication search**

Relying on the advances in natural language processing algorithms, the NLP Toolkit^[[1]](#footnote-1)^ (Zdravevski et al., 2019) was used to ensure an efficient search of the literature corpus. The NLP toolkit was designed to follow a formal method for search and assessment of the published studies in line with the rigorous principles of a systematic research (Moher et al., 2015). It automates the literature search, scanning, and eligibility assessment in the Preferred Reporting Items for Systematic Review and Meta-Analysis (PRISMA) methodological framework for systematic reviews (Moher et al., 2010). The scoping review methodology adopted in this study uses this framework to exploit an exact and transparent way of conducting a review, providing sufficient details to ensure the reproducibility of results. The workflow of a scoping review as proposed in (Arksey and O'Malley, 2005) includes five stages: (1) identification of a research question, (2) identification of relevant studies; (3) study selection, (4) charting the data; and (5) collating, summarizing, and reporting the results. The first three steps correspond to the PRISMA workflow steps: study collection, scanning, and eligibility evaluation. However, the final steps in a scoping review differentiate it from a systematic review for the lack of a qualitative analysis of identified papers and the number of studies involved (Arksey and O'Malley, 2005). Supplementary Figure 1 illustrates the number of identified, scanned, and included articles in this scoping review using the NLP toolkit.

#### Tuning the settings for the NLP Toolkit

To set up the NLP Toolkit for the review process a plan had to be developed that outlines decisions on which digital libraries will be queried, the relevant time span, suitable keywords, and properties that should be satisfied. The NLP toolkit ensures compliance with the terms of use of the digital libraries, with regard to the number of requests per unit time.

The NLP toolkit input parameters are a collection of keywords that are used to identify potentially relevant articles and a set of properties that should be satisfied by the identified articles. The input can be further expanded by proposing synonyms to the search keywords and properties either provided by the user or proposed by the toolkit and further fine-tuned if necessary.

*Keywords* are search terms or phrases that are used to query a digital library (e.g., “human microbiome and machine learning”, “human microbiome and artificial intelligence”). Eventual duplicates in the results are removed in a later phase. *Properties* are words or phrases that are being searched in the title, abstract or keywords section of the articles identified with the keywords. Examples of such properties employed in this study are “16S rRNA”, “metagenomic”, and “MAGs”. *Property groups* are thematically, semantically, or otherwise clustered properties for a more comprehensive presentation of the results. For example, the property group for the set of properties given in the example above can be “Input Data.” Supplementary Table 1 summarizes the relevant input categories used in this work.

*Start year* indicates the starting year of publishing (inclusive) for the papers to be included in the study. *End year* is the last year of publishing (inclusive) to be considered in the study. This automated search encompasses studies published from January 2008 to December 2019. *Minimum of the relevant properties* is a number denoting the minimum number of properties that an article must contain to be considered as relevant. In this study, this value was set to 3 and those were ([["human microbiome"], ["machine learning", "deep learning"]]), providing a right balance between falsely identifying relevant papers and potentially missing a relevant paper.

The manual search of digital libraries assumes using different search phrases, often involving complex Boolean conditions. The keywords described above are the NLP toolkit counterpart to these phrases. By screening the title and abstract, a reviewer determines whether the article is indeed relevant for the study. In the NLP toolkit, this process is automated using the properties and their synonyms to inspect if the information we are looking for is present in an article. The number of properties an article contains indicates its relevance for the performed review study. Undoubtedly, a human reader might better understand the context and better assess the relevance of an article. However, the NLP toolkit is mimicking these tasks, but in an automated and more thorough way, providing for an efficient and comprehensive scoping review process. For more information about the actual implementation, we refer the reader to the study by Zdravevski et al. (Zdravevski et al., 2019).

#### Identification of Relevant Studies

Upon the input categories were defined, the literature search was initiated using only the specified keywords to query the selected digital libraries. The NLP toolkit provides the support for the following digital libraries (i.e., sources): IEEE Xplore, Springer, and PubMed. It is worth noting that the NLP toolkit has used search engines of the corresponding publishers and retrieved their search results. Depending on the digital library in each search, the constraint on the number of retrieved articles differentiated. In the PubMed library, all articles matching the given search criteria were retrieved for further analysis. The IEEE’s search engine limits the number of articles in each search to 2000, all of which were retrieved. For Springer, the search for each keyword separately is limited to 1000 articles or 50 pages with results, whichever comes first, sorted by relevance determined by Springer.

#### Study Selection

The articles identified based on the specified keywords and retrieved from the publishers present an input to the study selection (i.e. screening and eligibility assessment) procedures. The results from numerous independent keyword-based searches were first merged, as some articles could be identified multiple times in the searches by different keywords or in multiple libraries. Therefore, the collected articles were screened, and their digital object identifier (DOI) was used to remove duplicates. In the screening process, the articles were further discarded if publication time does not correspond to the required time span, or if their title or abstract could not be analysed because of unavailability, parsing errors, or any other reason.

The study selection from the remaining set of articles exploits the advanced functionalities enabled by NLP tools. The NLP toolkit analyses a title and abstract for each study in an automated way, significantly reducing the number of articles for further manual screening. To automate the eligibility analysis several processing steps were followed: tokenization of sentences (Webster and Kit, 1992; Manning et al., 2014) and English stop words removal, stemming, and lemmatization (Manning et al., 2014) using the Natural Language ToolKit library (Bird et al., 2009). Stemmed and lemmatized properties were searched in the cleaned abstracts and titles, and each article was tagged with the detected properties.

The processed articles were labelled as relevant by the NLP toolkit if they contained at least three of the predefined properties in their title or abstract. To support the eligibility analysis, the selected articles were sorted by the number of identified property groups, number of identified properties, number of citations (if available), and year of publication, all in descending order. For the relevant articles, the toolkit automatically generated a bibliographic file (BibTeX) for simplified citations. The listing of the relevant articles identified by NLP toolkit based on three digital libraries: IEEE Xplore, PubMed, and Springer outputs the NLP toolkit as an Excel file with the following fields: the DOI, link, title, authors, publication date, publication year, number of citations, abstract, keywords, source, publication title, affiliations, number of different affiliations, countries, number of different countries, number of authors, BibTeX cite key, number of found property groups, and the number of found properties. This listing as an Excel file provides for any additional refined manual search of the articles with specific filtering criteria. The subset of targeted articles can subsequently be retrieved from their publisher and manually analysed for potential inclusion in the qualitative and quantitative synthesis.

#### Charting the Data

NLP toolkit can summarize and provide relevant statistics over the set of selected studies providing different type of charts. To support additional analyses of the research questions in this paper, a number of indicators were extracted from the selected studies. The trends in the application of ML methods in analysis of human microbiome over the observed publication period were analysed by aggregating the studies selected as relevant using several criteria: source (digital library) and relevance; selection criteria; publication year; digital library and publication year; search keyword and digital library; search keyword and year, property group and year; property and year, generating separate charts for each property group; and number of countries, the number of distinct affiliations and authors, aiming to simplify the identification of collaboration patterns. As an example, the chart presenting the distribution of the number of relevant articles in the period from January 2008 to December 2019 is presented in Supplementary Figure 2.

Besides the charts, the aggregated metrics can as well be exported in the form of comma-separated values files. The plotting of the aggregate results was integrated and streamlined using the Matplotlib library (Hunter, 2007) and NetworkX (Hagberg et al., 2008).

#### Number and Distribution of the Articles identified by the NLP toolkit

Using the NLP toolkit and searching 3 digital libraries: PubMed, IEEE Xplore and Springer, we identified 7,068 studies with potential relevance (Supplementary Figure 1). Duplicates that emerged in multiple independent searches were removed, reducing the total number to 5,935 studies. The first screening process further eliminated 4,928 studies published before the time interval of interest or for which the title or abstract could not be analysed for technical reasons, as described above. The remaining 1,007 studies underwent an automated eligibility assessment using the advanced NLP toolkit functionalities. After processing, the articles were tagged with the identified properties, and all articles containing less than 3 properties were removed. Overall, 67 articles were deemed eligible for further manual inspection. The obtained distribution revealed that IEEE Xplore, being a more technology-oriented library, had no relevant articles. PubMed contributed with 54 articles, while the Springer library contained 13 relevant papers.

### **Learning to rank model and microbiome GitHub repositories**

#### Data source: Microbiome research in the source code repositories

Researchers following the reproducible research movement tend to publish supporting resources like source code and data alongside the paper. One of the most popular choices for publishing supporting resources is GitHub, which provides hosting for software development version control using Git. While GitHub is a commercial platform, it supports open source projects by providing free hosting for them. Access to the source code supporting research allows for faster replication and validation of the results.

#### Identification of microbiome-related repositories on GitHub

During this study, 1,339 software repositories were identified in connection with microbiome research. A variety of use cases could be determined, including training materials and summer schools’ materials, tools, ongoing and published research. Additionally, several data sets were also found. All repositories were cloned, pre-processed, and tagged using simple keyword matching, and published at a public website [microbiome.przymus.org](../../../../C:/tmp/mozilla_eror0/microbiome.przymus.org). Initial estimates for different human microbiome-related repositories type count: Data - 115, Raw data - 18, Teaching materials – 83.

#### Research publications on GitHub

We extracted links and DOIs that linked repositories to research publications. This yielded a list of 4,327 publications, one repository contained 3,917 DOIs, and after manual check, it was excluded. Thus, 410 publications were used for further analysis. The identified DOIs intersected minimally with two other lists constructed for this task. We found less than 20 intersecting publications, showing that each of the approaches provides valuable materials.

#### Mining relevant research publications

In order to compile and maintain the list of the state-of-the-art publications and software, with the increasing trend of publications in this topic (see Supplementary Figure 2), it is crucial to select only the most relevant publications for further manual investigation. To provide for an efficient and reliable preselection process, we proposed an automated initial publication relevancy assessment model. The idea is to start with a small number of hand-picked publications and build a model that will be able to rank new publications according to the initial manual publications’ selection. We propose a semi-supervised learning as a ranking approach^[[2]](#footnote-2)^ for this problem.

##### Learning to rank and vector space model

Learning to rank is the process of applying ML to the ranking problem in information retrieval systems. The purpose of this process is to learn how to rank unseen documents or queries in a way that is similar to rankings made in the training data. The learning to rank approaches can be divided with respect to domain and codomain of the hypothesis function (Liu, 2009) into: pointwise, pairwise and listwise. Here, pointwise learning was used as a ranking approach. In the pointwise approach, the hypothesis function is defined based on feature vectors representing single documents, while the corresponding output is the relevance degree of a given single document (Liu, 2009). Thus, the hypothesis function can be modelled as regression, classification, or ordinal regression, with the corresponding loss function checking the accuracy of the prediction for each single document.

One common way to represent documents as features, is the vector space model, where a vector of terms represents the text. Each term becomes an independent dimension in a high dimensional vector space. Typically, terms are words, phrases or index identifiers (Singhal and Others, 2001). The terms present in a text get a non-zero value in the vector, depending on the predefined weight scheme.

##### Training dataset

The initial training dataset was constructed using a handpicked set of relevant publications. Additionally, we initially tagged a small subset of publications gathered from the GitHub repositories with tags “HUMAN” and “ML” based on keywords matched in the title. The structure of the initial dataset is as follows:

• manually-picked publications - 15 publications, – marked as “HUMAN” and “ML”,

• publications matched by the keywords in their title, – ML - 59 publications, – HUMAN - 93 publications.

Documents will be ranked based on how well they match the “HUMAN” and “ML” topics, based on the full text analysis.

##### Preprocessing

We followed a typical NLP processing pipeline:

1. Convert publications to pure text representation from PDFs and HTMLs;

2. Clean the text from the conversion artefacts and stop words;

3. Build the text corpus, with stemming and lemmatization;

4. Compute a TF-IDF representation for each document.

##### **Model** and scoring

The proposed model is a two-phase model that is heavily inspired by pointwise learning to rank approach presented in ref. (Fejzer et. Al, 2020):

The first phase of the used model is based on the feature importance analysis. First, using univariate feature selection, we select the 𝐾 best features based on univariate statistical tests. The statistical test estimates the degree of linear dependency, using the ANOVA F-Test between relevant and irrelevant documents on a given term. The goal is to select top terms that best distinguish the relevant publications from the irrelevant ones. Having selected the top 4 features, we assign them weights heuristically, according to the normalized F-value. Thus the score for the file is computed as

$f\left( q,d \right)=\sum_{k} w_{k}^{q}\cdot\phi_{k}\left( d \right)$ (1)

where $w_{k}^{q}$ is weight computed for term $k$, and $\phi_{k}\left( d \right)$ is the value for term $k$ in document $d\in D$.

The second phase uses augmented scoring to improve over scoring function results. This is done by adding the maximum of score to the score of relevant files

$f\left( q,d \right)=\sum_{i=1}^{K} w_{i}\cdot\phi_{i}\left( d \right)+1_{\left[ d\in relevant\left( q \right) \right]}\cdot\max_{i}f\left( q,d \right),$ (2)

where, $relevant\left( q \right)$ is a set of relevant scores for given topic $q$. Then augmented scoring is used as training target for regression model. This modification ensures proper training order, by ensuring that all relevant files have greater $f$ score than non-fixes. To create the target score we use a stochastic gradient descent regression model $f^{r}$ trained on the augmented score. The trained model $f^{r}$ is then used to compute the final scores for the documents.

For each publication, a score is computed using $f^{r}$ and a given topic:

- Human microbiome-related publications,
- ML related publications.

A higher score indicates higher resemblance to a given topic. To score publications both in ML and HUMAN topic, a combined score is used

$f^{r}\left( \text{Combined},d \right)=0.5\cdot f^{r}\left( ML,d \right)+0.5\cdot f^{r}\left( Human,d \right)$ (3)

##### Evaluation of results

The proposed method was evaluated comparing the computed scores with the labels (human expert evaluated) provided for a subset of articles. In particular, the first top 20 results were manually checked and all fit well into the given (“HUMAN” or/and “ML”) topics. Additionally, the first best scored 20 publications did not overlap with the publication lists yielded by other approaches used in this study. Improved accuracy is to be expected when the model is provided with more human-evaluated publications also including the negative examples. Basically, scoring catalogue new research publications require pre-processing of the document and getting the score from the trained model. For the purposes of this study, from the 410 identified papers based on keyword search, the proposed learning to rank approach preselected 29, out of which 17 were included in the final list after manual inspection.

It is worth noting that this approach is not limited just to the datasets obtained from GitHub.

# Supplementary Figures and Tables

## Supplementary Figures


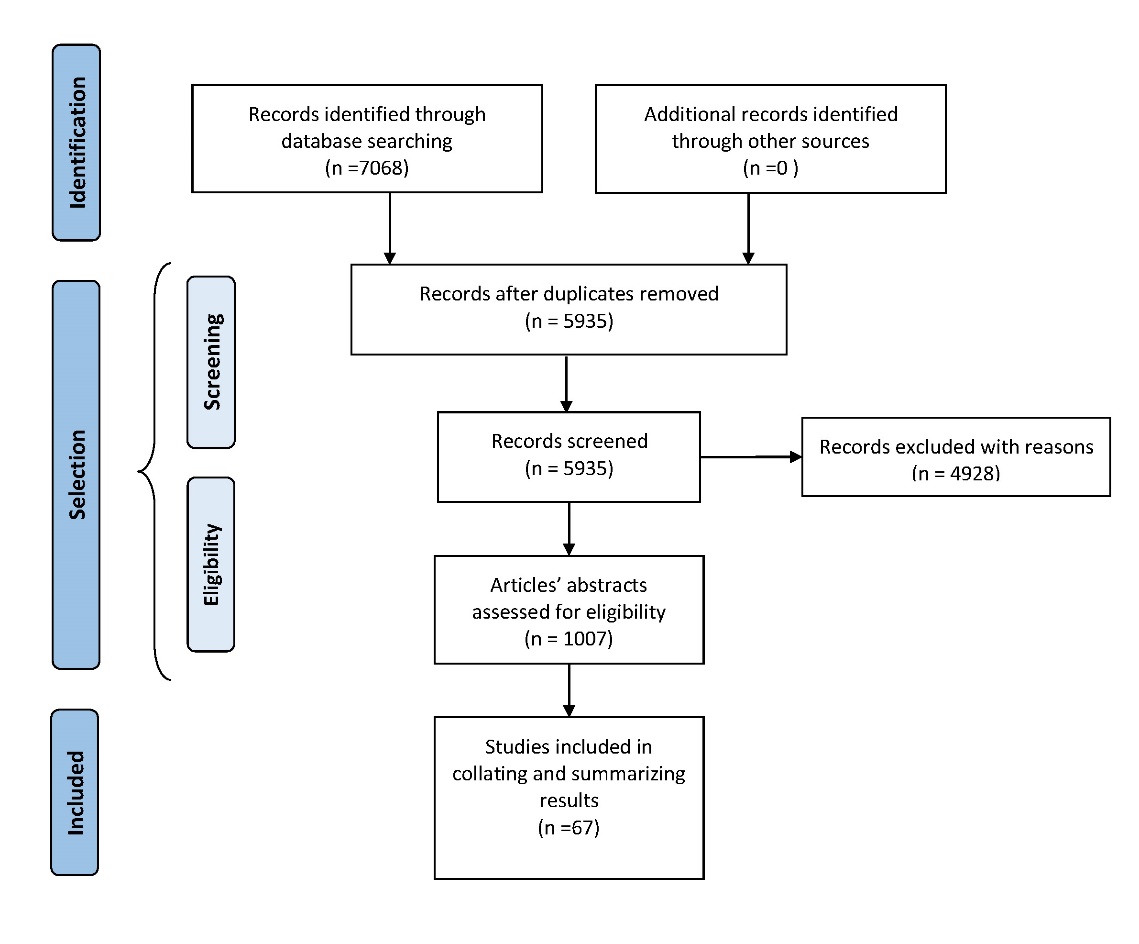


**Supplementary Figure 1.** The scheme presenting the scoping review workflow with details on paper identification process for the NLP Toolkit based search.


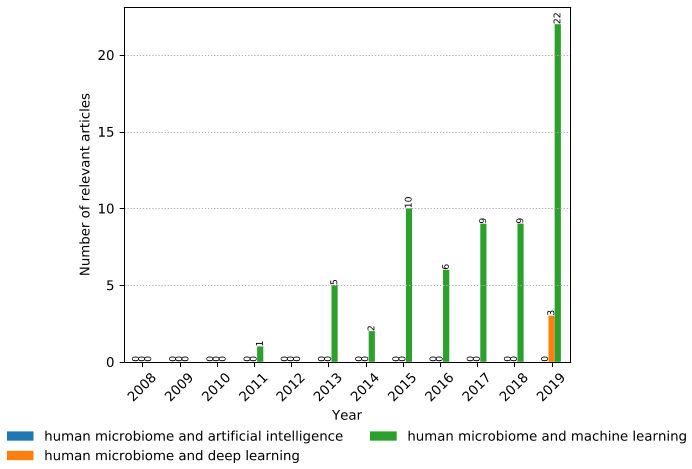


**Supplementary Figure 2.** The distribution of the number of relevant articles per year.

## **2.2. Supplementary Tables**

**Supplementary Table 1.** The natural language processing toolkit input parameters: keywords, properties, and property groups.

| **Property groups (properties)** | **Natural language processing toolkit input parameters** |
| --- | --- |
| Keywords | “human microbiome and machine learning", "human microbiome and deep learning", "human microbiome and artificial intelligence" |
| "Disease/Phenotype prediction" | ["infectious disease"], ["cardiovascular system disease"], ["vascular disease"], ["endocrine system disease"], ["gastrointestinal disease"], ["diarrhea"], ["gastroenteritis"], ["intestinal disease"], ["inflammatory bowel disease"], ["hematological disease"], ["immune system disease"], ["skin disease"], ["nervous system disease"], ["reproductive system disease"], ["respiratory system disease"], ["Inflammatory breast disease"], ["urinary tract disease"], ["neoplasm"], ["benign neoplasm"], ["cancer"], ["malignant tumour"], ["primary cancer"], ["pre-malignant neoplasm"], ["disease of mental health"], ["metabolic disease"], ["nutritional disorder"], ["nutritional deficiency disease"], ["overnutrition"], ["obesity"], ["syndrome"] |
| "Input Data" | ["16s rRNA"], ["OTU", "OTUs"], ["metagenomic"], ["MAGs"], ["Gene count", "Gene-pathway counts tables", "Gene counts tables", "pathway counts tables"] |
| “Methods" | ["supervised learning", "supervised methods", "supervised machine learning"], ["hierarchical multi-label classification", "hierarchical classification", "multi-label classification"], ["regression"], ["transfer learning"], ["time-series analysis", "time-series classification", "time-series prediction"], ["feature engineering"], ["feature construction", "feature extraction"],["Semi-supervised methods", "Semi-supervised learning"], ["link prediction"], ["unsupervised methods", "unsupervised learning", "unsupervised machine learning"], ["clustering"], ["dimensionality reduction", "feature selection"], ["network inference"], ["mathematical modelling"] |
| “Related with the study" | ["prediction"], ["disease prediction"], ["disease severity prediction"], ["treatment/side-effect prediction", "treatment prediction", "side-effect prediction"], ["patients stratification"], ["prognostic (remission/relapse)"], ["biomarker identification"], ["feature selection"], |
| "Resistance” | ["antimicrobial resistance"], ["antibiotic resistance"], ["treatment resistance", "drug resistance"], |
| "Taxonomic assignment" | ["taxonomic", "taxonomy"], ["metagenomic"], ["microbial"] |

# Supplementary References

Arksey H, and O'Malley L. (2005) Scoping studies: towards a methodological framework. Int J Soc Res Methodo. 8:1, 19-32. doi: 10.1080/1364557032000119616

Bird, S., Klein, E., and Loper, E. (2009). *Natural Language Processing with Python: Analyzing Text with the Natural Language Toolkit*. “O’Reilly Media, Inc.

Fejzer M, Narębski J, Przymus P, and Stencel K (2020) Tracking Buggy Files: New Efficient Adaptive Bug Localization Method. Preprint, 2020

Hagberg, A., Swart, P., and S Chult, D. (2008). Exploring network structure, dynamics, and function using NetworkX. Los Alamos National Lab (LANL), Los Alamos, NM (United States).

Hunter, J. D. (2007). Matplotlib: A 2D Graphics Environment. *Comput. Sci. Eng.* 9, 90–95. doi: 10.1109/MCSE.2007.55.

Liu, T.-Y. (2009). Learning to Rank for Information Retrieval. *Foundations and Trends® in Information Retrieval* 3, 225–331.

Manning, C., Surdeanu, M., Bauer, J., Finkel, J., Bethard, S., and McClosky, D. (2014). The Stanford CoreNLP Natural Language Processing Toolkit. in *Proceedings of 52nd Annual Meeting of the Association for Computational Linguistics: System Demonstrations* (Stroudsburg, PA, USA: Association for Computational Linguistics), 55–60

Moher, D., Liberati, A., Tetzlaff, J., Altman, D. G., and PRISMA Group (2010). Preferred reporting items for systematic reviews and meta-analyses: the PRISMA statement. *Int. J. Surg.* 8, 336–341.

Moher, D., Stewart, L., and Shekelle, P. (2015). All in the Family: systematic reviews, rapid reviews, scoping reviews, realist reviews, and more. *Systematic Reviews* 4. doi:10.1186/s13643-015-0163-7.

Singhal, A. (2001). Modern information retrieval: A brief overview. *IEEE Data Eng. Bull.* 24, 35–43.

Webster, J. J., and Kit, C. (1992). Tokenization as the initial phase in NLP. in *COLING 1992 Volume 4: The 15th International Conference on Computational Linguistics*.

Zdravevski, E., Lameski, P., Trajkovik, V., Chorbev, I., Goleva, R., Pombo, N., et al. (2019). Automation in Systematic, Scoping and Rapid Reviews by an NLP Toolkit: A Case Study in Enhanced Living Environments. *Lecture Notes in Computer Science*, 1–18. doi:10.1007/978-3-030-10752-9_1.

1. For source code of the toolkit and the resulting report see <https://gitlab.com/magix.ai/article-analysis-public> and <https://gitlab.com/magix.ai/survey-results>, respectively. [↑](#footnote-ref-1)
2. For proof-of-concept code see https://github.com/przymusp/human_microbiome_learn_to_rank_papers. [↑](#footnote-ref-2)
